# The Neural Correlates of Chewing Gum—A Neuroimaging Review of Its Effects on Brain Activity

**DOI:** 10.3390/brainsci15060657

**Published:** 2025-06-18

**Authors:** James Chmiel, Agnieszka Malinowska

**Affiliations:** 1 Institute of Physical Culture Sciences, Faculty of Physical Culture and Health, University of Szczecin, Al. Piastów 40B Block 6, 71-065 Szczecin, Poland; 2 Doctoral School of the University of Szczecin, University of Szczecin, Mickiewicza 16, 70-384 Szczecin, Poland; 3 Institute of Psychology, University of Szczecin, 71-017 Szczecin, Poland

**Keywords:** chewing gum, gum chewing, chewing, EEG, electroencephalography, electroencephalogram, fMRI, fNIRS, neuroimaging, QEEG, quantitative electroencephalography

## Abstract

Introduction: Chewing gum is a widespread, seemingly mundane behaviour that has been linked to diverse benefits such as improved cognitive performance, reduced stress, and enhanced alertness. While animal and human research indicate that mastication engages extensive sensorimotor networks and may also modulate higher-order cognitive and emotional processes, questions remain about the specific neural mechanisms involved. This review combines findings from neuroimaging studies—including fMRI, fNIRS, and EEG—that investigate how chewing gum alters brain activity in humans. Methods: Using a targeted search strategy, we screened the major databases (PubMed/Medline, Scopus, ResearchGate, Google Scholar, and Cochrane) from January 1980 to March 2025 for clinical studies published in English. Eligible studies explicitly measured brain activity during gum chewing using EEG, fNIRS, or fMRI. Results: After a title/abstract screening and a full-text review, thirty-two studies met the inclusion criteria for this review: 15 utilising fMRI, 10 using fNIRS, 2 using both fNIRS and EEG, and 5 employing EEG. Overall, the fMRI investigations consistently reported strong activation in bilateral motor and somatosensory cortices, the supplementary motor area, the insula, the cerebellum, and the thalamus, during gum chewing, with several studies also noting involvement of higher-order prefrontal and cingulate regions, particularly under stress conditions or when participants chewed flavoured gum. The fNIRS findings indicated that chewing gum increased oxygenated haemoglobin in the prefrontal cortex, reflecting heightened cortical blood flow; these effects were often amplified when the gum was flavoured or when participants were exposed to stressful stimuli, suggesting that both sensory and emotional variables can influence chewing-related cortical responses. Finally, the EEG studies documented transient increases in alpha and beta wave power during gum chewing, particularly when flavoured gum was used, and reported short-lived enhancements in vigilance or alertness, which tended to subside soon after participants ceased chewing. Conclusions: Neuroimaging data indicate that chewing gum reliably engages broad sensorimotor circuits while also influencing regions tied to attention, stress regulation, and possibly memory. Although these effects are often short-lived, the range of outcomes—from changes in cortical oxygenation to shifts in EEG power—underscores chewing gum’s capacity to modulate brain function beyond simple oral motor control. However, at this time, the neural changes associated with gum chewing cannot be directly linked to the positive behavioural and functional outcomes observed in studies that measure these effects without the use of neuroimaging techniques. Future research should address longer-term impacts, refine methods to isolate flavour or stress variables, and explore potential therapeutic applications for mastication-based interventions.

## 1. Introduction

Mastication, commonly understood as the act of chewing, is far more than a mechanical process for breaking down food. A growing body of research indicates that chewing has profound effects on various physiological and cognitive functions, underscoring its importance in overall health. For instance, several studies have highlighted that the rhythmic act of chewing activates extensive areas of the brain, including those in the somatosensory cortex, motor cortex, thalamus, and cerebellum, and it also enhances regional cerebral blood flow [1,2]. As a result, mastication helps maintain the integrity and function of regions critical for higher cognitive processes, such as the hippocampus and prefrontal cortex [3,4]. These brain areas support processes like learning, memory, and executive function—illustrating why the simple habit of chewing plays a role in both physical and mental well-being.

Researchers have also explored how chewing influences the hypothalamic–pituitary–adrenal (HPA) axis, a central stress response system. Studies in animals show that masticatory stimulation can help mitigate the hyperactivity of the HPA axis by controlling glucocorticoid levels, the body’s key stress hormones [1,3]. This regulation not only helps mitigate the adverse effects of chronic stress on the brain but also enhances cognitive performance and emotional stability. In contrast, prolonged masticatory dysfunction—whether due to tooth loss, occlusal disharmony, or a habitual soft diet—can elevate stress-hormone levels that, over time, may induce changes in the hippocampus linked to learning and memory deficits [1,4,5]. These findings underscore that proper mastication helps maintain balanced neuroendocrine responses and optimises the brain’s capacity to adapt to stress.

In parallel, human studies have underscored the relationship between robust masticatory function and healthy cognitive ageing. Research suggests that older adults who retain more natural teeth or use well-fitted prostheses to simulate natural chewing experience better outcomes in episodic memory and overall cognition [6]. By contrast, diminished chewing efficiency—often arising from tooth loss, ill-fitting dentures, or problems with the temporomandibular joint—has been linked to decreased activity in the prefrontal cortex and hippocampus, impairing tasks related to memory consolidation, spatial learning, and executive decision-making [1,4]. Adequate rehabilitation of the occlusion, for instance through dental restorations or prosthetic appliances, may help restore a level of chewing force sufficient to stimulate neural circuits and preserve cognitive function [5,6].

Chewing gum is a widely practiced oral activity, often used to promote oral hygiene. Numerous studies demonstrate that regular gum chewing enhances oral health [7,8]. Beyond these benefits, emerging evidence suggests that it may also affect brain function, particularly cognitive processes. For instance, some studies indicate that gum chewing improves immediate knowledge recall [9,10], while others show it facilitates delayed memory retention [11,12,13,14,15].

Furthermore, mastication appears to function as a “natural stress-coping mechanism” [16] that not only dampens anxiety [17,18] but also enhances alertness [19,20,21,22] and concentration [23]. During cognitively demanding tasks—such as academic testing or during heightened anxiety—chewing gum or other chewable materials has been associated with improvements in focus, reduction in psychological stress markers, and increased subjective calmness [24,25]. Although the precise biological underpinnings still warrant further research, many scientists point to simultaneous increases in cerebral blood flow, reduced cortisol release via the HPA axis, and peripheral somatosensory feedback from the masticatory muscles. Overall, chewing during stressful or demanding situations may provide an accessible, non-pharmacological strategy approach to enhance mental clarity and resilience.

Given that every cognitive, pathophysiological, and behavioural state is reflected in corresponding brain activity, it is hypothesised that the diverse behavioural and functional effects of gum chewing are detectable through neuroimaging techniques. Modern neuroscience offers various tools, including electroencephalography (EEG), functional near-infrared spectroscopy (fNIRS), and functional magnetic resonance imaging (fMRI), to explore brain function. This mechanistic review synthesises findings from EEG, fNIRS, and fMRI studies to elucidate the neural mechanisms and regional brain activations linked to gum chewing in humans. Furthermore, this study aims to establish a relationship between the observed beneficial effects of gum chewing, as measured by various assessments, and the concomitant changes in brain activity.

## 2. Methods

This mechanistic review aims to systematically evaluate neuroimaging research on gum chewing. Strict selection criteria and a comprehensive literature search were utilised to ensure the validity and relevance of the included evidence. This review targeted original empirical studies—encompassing observational and experimental designs—that investigated the effects of gum chewing on brain activity. The approach partially adopted established practices for systematic reviews and evidence synthesis, such as PRISMA. However, as a mechanistic review focused on elucidating the neural correlates and brain mechanisms of gum chewing using EEG, fMRI, and fNIRS studies, it does not fully conform to PRISMA standards for systematic reviews. Consequently, it excludes a formal PICOS evaluation and Risk of Bias assessment. For this reason, we did not register this study in the PROSPERO database.

### 2.1. Data Sources and Search Strategy

The following set of combined keywords was used by J.Ch. and A.M. in their independent, standards-compliant Internet search for this review: “EEG” OR “QEEG” OR “electroencephalogram” OR “electroencephalography” OR “fMRI” OR “functional magnetic resonance imaging” OR “functional near-infrared spectroscopy” OR “fNIRS” AND “gum chewing.” With a focus on papers published between January 1980 and March 2025, a thorough search was conducted in March 2025 utilising a number of databases, including PubMed/Medline, Scopus, Research Gate, Google Scholar, and Cochrane.

### 2.2. Study Selection Criteria

Clinical trials published in English between January 1980 and March 2025 that explicitly examined brain activity using EEG, fMRI, and fNIRS whilst chewing gum were required for inclusion in this review. Review articles or publications not written in English were excluded. The studies sought for this review may or may not have a control group.

### 2.3. Screening Process

To ensure that pertinent research was included and studies that did not meet predetermined criteria were excluded, a systematic screening procedure was implemented. Two independent reviewers, J.Ch. and A.M., thoroughly reviewed abstracts and titles during the initial screening process.

#### 2.3.1. Title and Abstract Screening

To decide which studies met the inclusion requirements, each reviewer assessed the abstracts and titles of the publicly available records. The focus of the screening criteria for gum chewing EEG, fMRI, and fNIRS results.

#### 2.3.2. Full-Text Assessment

Publications that passed the abstract and title screening underwent a thorough full-text review. Reviewers carefully assessed each study to ensure it met the eligibility criteria, focusing on clinical trials published in English between January 1980 and March 2025.

## 3. Results

**Figure 1:**
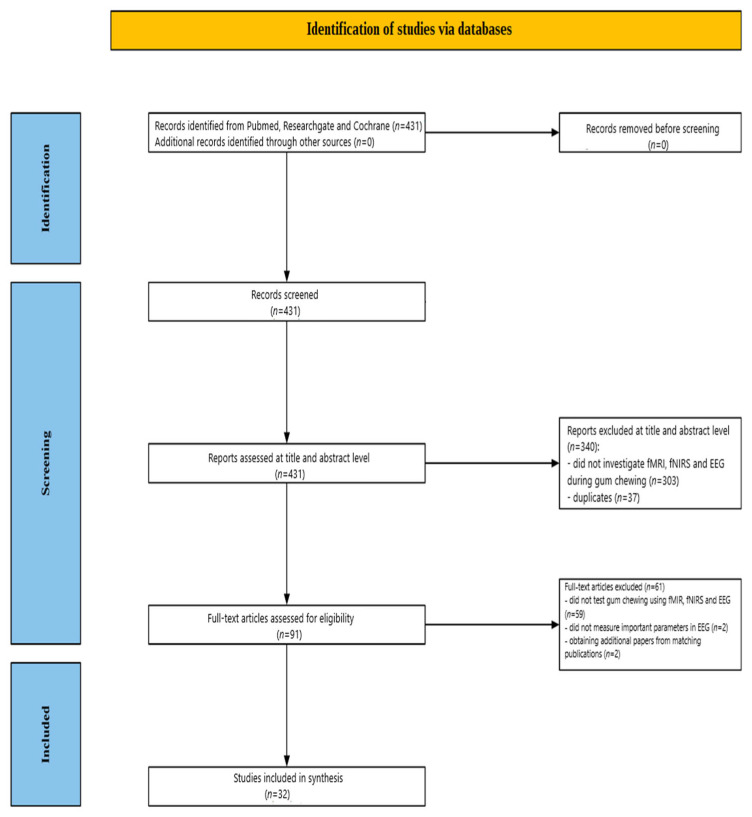
Flowchart depicting the different phases of the systematic review.

Figure 1 illustrates the screening process. Initially, 431 studies were identified through database searches. Of these, 340 publications were excluded after a review of their abstracts and titles: 303 did not investigate EEG, fMRI, and fNIRS during gum chewing, and 37 were duplicates. Following a detailed full-text review of the remaining 91 papers, 59 studies were further excluded for not addressing EEG, fMRI, and fNIRS during chewing gum. Two EEG studies were excluded for not measuring key EEG parameters during gum chewing Additionally, two fMRI studies were included, after being identified through searches for matching publications. Ultimately, 32 studies were included in the review: 15 fMRI, 12 fNIRS, and 5 EEG, published between 1998 and 2024.

### 3.1. Summary of Included Studies

#### 3.1.1. fMRI Studies

The 15 included fMRI studies [26,27,28,29,30,31,32,33,34,35,36,37,38,39,40] are summarised in Table 1. Collectively, these investigations examined how chewing gum—or, more broadly, masticatory behaviour—modulates brain activation. Despite varying in specific objectives, participant demographics, chewing protocols, and analytical methods, the studies converge on several key findings: (1) chewing reliably engages a broad sensorimotor network, (2) masticatory tasks often extend beyond primary motor regions to involve higher-order cortical areas and subcortical structures, and (3) individual factors such as chewing-side preference, aging, and bolus hardness influence neural responses to chewing.

##### Chewing Paradigms Employed Across the fMRI Literature

Although every experiment contrasted an active masticatory phase with a quiescent baseline, the behavioural methods used to induce chewing were far from uniform. A close reading of the protocols reveals that the studies coalesce into six functional families, each designed to address a distinct neurobiological question.

First, the “canonical rhythmic-chewing” family—represented by the foundational mapping studies [26], the ageing paper [38], and the large ICA dataset [39]—instructed volunteers to chew tasteless, odourless gum at approximately one cycle per second for 20-to-32 s epochs, repeated three-to-ten times. These paradigms served as the field’s baseline for characterising a “typical” chewing network and were occasionally modified to test hardness effects (moderately hard versus very hard gum in [26]) or lifespan changes (young, middle-aged, elderly in [38]).

A second group deliberately manipulated mechanical load. In both [26,33], identical block designs were executed twice, once with a soft bolus and once with a hard one (a wine-gum in [33]). The aim was to ascertain whether the amplitude or topography of brain activity scales with bite force and bolus texture. Indeed, both papers observed a cortical–cerebellar trade-off: harder material dampened MI/SI BOLD yet heightened cerebellar responses.

Third, several studies probed laterality and habitual chewing side. Volunteers either chewed exclusively on the right or left with the aid of a rubber-dam strip [30], used their naturally preferred side [27], or undertook a single one-hour unilateral chewing bout to examine post-exercise perfusion changes in the trigeminal nucleus [40]. These paradigms aimed to determine whether hemispheric activation merely follows the bolus location or instead reflects long-standing behavioural preferences. Results converged on the latter: right-handers assigned to chew on the non-dominant side exhibited nearly symmetrical maps, whereas individuals who habitually initiated chewing on the left displayed true contralateral dominance.

A fourth set layered chewing onto a second motor or cognitive demand. In the dual-task study [28], subjects performed right index flexion and extension concurrently with chewing; in [35], they performed the Attentional Network Test within the scanner, with and without gum. These designs quantified how orofacial activity shares neural resources with unrelated hand-motor or attentional systems. The principal finding was that concurrent chewing diverts resources away from contralateral S1 yet, paradoxically, shortens overall reaction time by recruiting premotor and cingulo-frontal control hubs.

Finally, two studies embedded chewing within affective or sensory contexts. The noise-stress protocol [34] involved participants chewing continuously during the delivery of 90 dB white noise bursts, revealing that masticatory activity dampens left anterior-insula stress encoding and decouples it from the dACC. The mint-experience study [36] paired chewing with monorhinal odour presentation (trigeminal menthol vs. non-trigeminal carvone). Frequent gum users exhibited heightened mid-cingulate and SMA responses to trigeminal input.

Despite this methodological diversity, the paradigms converge on three shared technical features. First, all employed a block structure with alternating on–off epochs, typically 25 s long, except for the dynamic experiment [31], which subdivided each chew block into five 5 s bins to track intra-block evolution. Second, pacing was consistently maintained at approximately 1 Hz, enforced by a metronome in eleven of fifteen studies; the remainder permitted self-paced chewing after a pre-chew period to stabilise the bolus. Third, rest conditions were sensory-matched: participants either held the gum motionless in one cheek or maintained light centric occlusion, thereby preserving tactile context while eliminating rhythmic proprioception.

##### Participants Characteristics

Across the 15 eligible studies [26,27,28,29,30,31,32,33,34,35,36,37,38,39,40], a total of 291 healthy volunteers were examined (mean group size = 19.4 ± 10.1; range = 8–38). Ages spanned from 19 to 73 years, with most cohorts concentrated in young adulthood (20–35 years). One study deliberately sampled three age-bands to capture ageing effects [38]. Sex ratios were balanced overall (≈55% male), and all but two projects restricted inclusion to right-handed subjects; the remaining trials compared naturally right- and left-handed individuals [33] or stratified by unilateral chewing preference [27,40]. All participants were dentate, free of neurological, psychiatric, or temporomandibular disorders and, where relevant, abstained from caffeine, alcohol, or flavoured foods prior to scanning.

##### fMRI Methodologies

- Scanner Hardware and Sequences

The chewing experiments were performed on routine clinical MRI systems operating at two field strengths: eight studies collected data on 1.5-tesla units [26,28,30,32,33,34,38,40] and seven on 3-tesla units [27,29,31,35,36,37,39]. Irrespective of field strength, functional images were obtained with T2*-weighted gradient-echo echo-planar imaging optimised for the blood-oxygenation-level-dependent (BOLD) contrast; only study 40 added a pseudocontinuous arterial-spin-labelling sequence to quantify resting perfusion before and after the chewing exercise. Acquisition parameters clustered within a narrow range that balanced brain coverage against the inevitable jaw motion. At 1.5 T, repetition times centred on about 4 s (3.0–4.0 s) with echo times around 44 ms, whereas the 3 T protocols used shorter repetition times close to 3 s (2.0–3.0 s) and echo times of roughly 30 ms. All protocols kept a 90° flip angle, a 64 × 64 phase-encoding matrix and whole-brain coverage extending through the cerebellum and brainstem. In-plane pixel dimensions averaged 3.5–3.8 mm at 1.5 T and 3.0–3.5 mm at 3 T; slice thickness lay between 3.0 and 3.8 mm, usually separated by a 0–0.5 mm inter-slice gap, yielding voxel volumes of about 50 mm^3^ that afford a favourable signal-to-noise ratio while tolerating small chewing-related displacements. Every study also acquired a high-resolution three-dimensional T1-weighted anatomical scan (typically MPRAGE or IR-FSPGR, 1 mm isotropic) for accurate co-registration and spatial normalisation of the functional data.

- Task Design

All fifteen investigations adopted a block design in which rhythmic mastication alternated with rest, yet each study tuned specific behavioural parameters to interrogate different facets of oro-facial control. Chewing epochs typically lasted 20–32 s (median ≈ 25 s) and were repeated three to ten times per run, producing functional series of roughly 4–7 min; the sole outlier was the perfusion experiment that inserted a single 60 min chewing bout between two pCASL scans [40]. Jaw cycles were paced by a metronome at ≈1 Hz in most protocols [26,27,28,29,30,31,32,34,35,36,37,38,39], ensuring regular motion while remaining comfortable inside the head-coil. Gum was almost always tasteless and odourless and, where necessary, pre-chewed outside the scanner to stabilise consistency and bite force [31,34].

Investigators then over-laid targeted manipulations. Mechanical load was varied by comparing moderately hard with very hard gum [26] or by contrasting soft chewing gum with tougher wine-gums [33]. Laterality was explored by forcing the bolus onto one side with rubber-dam strips [30], asking participants to chew exclusively on the right [31] or left/right according to individual chewing-side preference [27], or by sustaining unilateral chewing for an hour to probe vascular plasticity [40]. Handedness effects were examined directly in a cohort of right- versus left-handers during unconstrained chewing [33].

To test dual-task interference, one study combined mastication with right-hand finger flexion–extension [28], while another embedded chewing within the Attentional Network Test to assess alerting and executive control [35]. Emotional modulation was addressed by pairing chewing with bursts of 90 dB white-noise and sampling block-wise stress ratings [34]. In the search for a putative masticatory central-pattern generator, participants performed spontaneous chewing, deliberately slow “controlled” chewing, and a non-orofacial rhythmic hand task (rosary-bead pulling) within the same session [37].

Rest blocks were standardised across studies: subjects either kept the mandible motionless with the bolus parked passively in the cheek or maintained light centric occlusion of the teeth, thereby matching tactile context while eliminating cyclical proprioceptive input [26,27,28,29,30,31,32,33,34,35,36,37,38,39,40].

- Motion control and preprocessing

Because rhythmic jaw motion can generate substantial head displacement and susceptibility artefacts, every study implemented a two-tier strategy of physical stabilisation followed by aggressive post-acquisition cleanup. Subjects’ heads were secured with combinations of vacuum cushions, Velcro straps, foam wedges, or bite-bars [26,30,31,37], and tasks were rehearsed to maintain a comfortable 1 Hz rhythm that minimised parasitic movements [28,32]. Five groups monitored jaw or masseter EMG in real time, cancelling and repeating runs that breached preset amplitude windows [31,34,40].

During reconstruction, most teams discarded the first 3–8 volumes to allow for T1-equilibrium [26,27,28,29,30,31,32,34,35,36,37,38,39] and rejected whole runs if peak translation exceeded 0.75–1.5 mm or rotation surpassed 0.5–1° [26,27,30,33,38]. Residual volumes were realigned with six-parameter rigid-body correction in SPM 99/5/8/12 [27,28,29,31,32,34,35,36,37,38,39] or in AIR 3.0 plus a secondary SPM pass [26,38]; BrainVoyager’s sinc-interpolated realignment was used once [30]. To attenuate chewing-synchronous micromovements that survive rigid correction, two studies applied a low-pass temporal filter at 1.5 s within MEDx, driving residual displacement below 0.01 mm [26,38].

After motion correction, functional series were co-registered to each participant’s T1 anatomical volume, normalised to MNI space via affine and non-linear warps, and smoothed with a 6–8 mm FWHM Gaussian kernel to accommodate inter-subject variability (all studies). Slice-timing correction preceded realignment when TR ≤ 3 s [29,31,35,36]. Global intensity drifts were removed either by proportional scaling [26,38] or by including the six motion vectors and their temporal derivatives as nuisance regressors in the general linear model [28,34,35].

For advanced artefact control, study 40 rigidly co-registered pre- and post-exercise pCASL images to identical brain-stem voxels and limited analyses to volumes with <10% rejected TRs, while the ICA trial [39] decomposed residual noise components before back-reconstruction. Collectively, these procedures contained chewing-related motion to sub-voxel levels, permitting reliable estimation of the blood-oxygenation or perfusion response during mastication.

##### Canonical Chewing-Related Activation Pattern

Despite wide variation in scanner platforms, paradigms, and analytic pipelines, every study converged on a remarkably consistent bilateral cortico-subcortical network that is recruited whenever humans rhythmically chew. The core cortical focus lies around the orofacial representations of the pre- and post-central gyri (Brodmann areas 4 and 3). Activation spreads ventrally into the operculum and posterior insula, reflecting the fusion of efferent motor drive with rich intra-oral somatosensory feedback [26,27,28,29,30,31,32,33,37,38,39]. Forward from the central sulcus, activity ascends onto the supplementary and pre-motor cortices (dorsal and ventral BA 6), regions linked to sequencing and timing of repetitive movements [26,31,32,35,37].

Subcortically, chewing invariably excites the thalamus—the principal relay for trigeminal and proprioceptive afference—and the basal ganglia, most often the putamen and head of caudate, which together orchestrate automatic movement initiation and gain control [27,31,37,39]. The cerebellum shows two-tier involvement: the anterior lobe scales bite force and muscle synergies, whereas more lateral posterior zones couple chewing to cognitive and affective contexts [26,30,31,32,37,38,39].

Although technical obstacles limit brain-stem sensitivity, several groups still detected task-locked responses in the trigeminal principal nucleus, substantia nigra, and pontine nuclei—structures long posited to house rhythm-generating circuits [27,31,37,40]. Group-ICA further demonstrated that these motor elements are inter-digitated with a fronto-cingulo-parietal network engaged in attention, working memory, and emotion, implying that mastication is monitored by executive systems even when behaviour is over-learned [32,34,35,39].

In sum, human mastication is not a simple brain-stem reflex but a distributed, hierarchically organised process that recruits primary sensorimotor territories, premotor sequencing hubs, thalamo-basal-cerebellar loops, and higher-order cognitive–affective nodes in a tightly coupled bilateral ensemble.

##### Modulators of Brain Activation

The canonical chewing network is malleable: its amplitude, laterality, and anatomical spread fluctuate with mechanical load, task context, affective state, lifetime habit, and ageing.

- Mechanical load

When subjects chewed a moderately hard gum (“X”), the BOLD rise in the primary sensorimotor cortex, SMA, and insula was 8–22% larger than when they chewed a very hard gum (“G”); on the left MI, for example, X-gum evoked a 100% response that fell to 84% with G-gum (F_1,26_ = 8.44) [26]. The pattern reversed in the cerebellum, where the harder bolus produced a 29–41% stronger signal (left cerebellum F_1,26_ = 41.15) [26]. A complementary experiment using soft chewing gum versus hard wine-gum replicated the effect: the softer bolus recruited 17% more cortical voxels overall and yielded hemisphere-dominant MI activation in right-handed volunteers [33].

- Laterality, handedness, and chewing-side preference

In individuals who habitually begin mastication on the left, rhythmic chewing preferentially activated the right MI/SI and right IFG, whereas right-side preference showed the mirror pattern (left MI/SI) [27]. Yet, when right-handed adults were forced to chew unilaterally with a rubber dam, activation remained strikingly symmetrical: the lateralisation index never exceeded 0.055 and MI peak coordinates differed by <3 mm between right- and left-chew runs [30]. True hemispheric dominance therefore appears to reflect ingrained behavioural preference rather than the transient side of the bolus. Consistent with this, whole-brain maps flipped with handedness: right MI/SMA prevailed in right-handers, and left MI/SMA in left-handers during unconstrained gum chewing [33].

- Concurrent motor or cognitive load

Coupling mastication with 1 Hz finger flexion–extension reduced contralateral SM1 recruitment from 2090 to 1296 voxels (−38%) and shrank S1 activation three-fold (718 → 271 voxels) [28]. During the Attentional Network Test, chewing shortened mean reaction time by 36 ms (523 ± 62 ms vs. 559 ± 80 ms, *p* < 0.001) and shifted the neural focus from cerebellum to premotor and cingulo-frontal areas: BA 6 and ACC showed larger responses, whereas the cerebellar anterior lobe was suppressed [35]. Thus, mastication competes with primary sensorimotor resources yet facilitates higher-order control when cognition is engaged.

- Affective context

Exposure to 90 dB white-noise elevated subjective stress (SVAS-20 = 56 ± 20) and drove the left anterior insula (AI) and superior temporal sulcus (STS); chewing blunted both effects (stress = 45 ± 18; AI noise × chew interaction F_1,15_ = 5.05; STS F_1,15_ = 15.72) [34]. Psychophysiological interaction and dynamic-causal-modelling analyses revealed that gum chewing weakened noise-evoked AI ↔ dACC coupling and reversed the STS → AI information flow (β = −0.70 Hz vs. + 0.28 Hz when not chewing), indicating a dual action on sensory encoding and interoceptive appraisal [34].

- Ageing

Chewing-induced BOLD amplitude in MI/SI, thalamus, and anterior cerebellum declined by ~37% in middle age and ~67% in older adults relative to young controls (sensorimotor cortex 63.3% and 32.7% of youthful levels) [38]. In contrast, right prefrontal recruitment escalated more than four-fold with age (174% in middle-aged, 413% in elderly; *p* < 0.0001), while SMA and insular responses remained stable, suggesting compensatory top-down engagement as sensorimotor efficiency wanes [38].

- Habitual mint-gum use

Frequent chewers (≥2 × week) lateralised a trigeminal menthol puff to the correct nostril 21% more often than infrequent chewers (F_1,25_ = 13.85) and showed stronger activation in the mid-cingulate, SMA, and postcentral gyrus for L-menthol > L-carvone (right SMA 6 cm^3^ cluster, peak t = 4.12). Chewing frequency correlated with lateralisation ability (r = 0.64, [36], implying experience-dependent plasticity of trigeminal circuits.

- Prolonged mastication

After a single 60 min chewing bout, the ipsilateral trigeminal principal nucleus perfusion rose from 30.2 ± 1.8 to 39.4 ± 2.4 mL min^−1^ 100 g^−1^ (+30%) while the dorsolateral mid-brain was unchanged; the magnitude of change scaled with individual chewing-side preference (R^2^ = 0.23) [40].

Collectively, these modulators show that the chewing network is not hard-wired: its sensorimotor core flexes with bite force and dual-task competition, its hemispheric balance mirrors durable behavioural asymmetries, its limbic fronto-insula arm dampens external stress when the jaw is active, and its responsiveness is sculpted both by decades of ageing and by months or years of mint-gum habit.

#### 3.1.2. Network-Level Organisation

Beyond the regional activations described above, several groups examined how the chewing nodes interact as distributed functional networks. The most comprehensive account comes from a Group-ICA decomposition of 38 young adults that yielded three task-related independent components. IC 1 (r = 0.876 with the chewing regressor) represented a “classic” sensorimotor loop linking bilateral anterior cerebellum, pontine/midbrain nuclei, thalamus, putamen, insula, and peri-Rolandic cortex—essentially the machinery for sensorimotor integration and force modulation [39]. IC 2 (r = 0.74) mapped onto a cognitive–emotional circuit spanning the lateral cerebellar hemispheres (“cognitive cerebellum”), caudate, sub-/pre-genual anterior cingulate, dorsolateral and ventrolateral prefrontal cortices and inferior/superior parietal lobules [39]. IC 3 (r = 0.668) reflected syntax/visual-instruction processing (inferior frontal gyrus, thalamus, caudate), illustrating that ICA is sensitive enough to segregate task-irrelevant yet stimulus-driven ensembles [39].

Dynamic approaches corroborated this multi-network view. During the attentional-network test, chewing shifted effective connectivity from a cerebellar-dominant motif at rest to a premotor/ACC-centred motif when cognitive control was demanded [35]. In the acoustic-stress paradigm, psychophysiological interaction analysis showed that noise strengthened coupling between the anterior insula and dorsal ACC, but gum chewing short-circuited this pathway, reducing the insula’s access to the salience network and dampening subjective stress; dynamic-causal modelling pinpointed the mechanism as an attenuation of STS → insula drive (β = −0.70 Hz vs. +0.28 Hz when not chewing) [34].

Efforts to localise a human masticatory central-pattern generator (CPG) found a cerebellar epicentre: a conjunction contrast between spontaneous chewing and a rhythmic hand task isolated a supero-medial cerebellar cluster that was silent during slow, voluntary jaw openings, implying rhythm-generation rather than generic motor output [37]. Brain-stem responses in the trigeminal principal nucleus and substantia nigra [27,31,40] suggest additional sub-cortical CPG contributors, but susceptibility artefacts currently hamper precise mapping.

Taken together, the evidence indicates that human mastication is embedded in at least three hierarchically coupled systems: (i) a core sensorimotor loop for pattern execution, (ii) a fronto-cingulo-parietal control network that monitors, times, and contextualises the movement, and (iii) a limbic–salience interface that modulates interoceptive and affective significance. Chewing therefore represents a paradigmatic behaviour where low-level rhythmic generators interface seamlessly with high-level cognitive–emotional circuits.

#### 3.1.3. Temporal Dynamics of the Chewing Cycle

Only one study modelled chewing at a sub-block time scale, yet it provides a detailed picture of how the masticatory network waxes and wanes across a single bout. Onozuka and colleagues divided each 25 s chewing epoch into five consecutive 5 s bins and entered them as separate regressors in the general linear model. The resulting contrasts revealed a triphasic profile.

The first is the initiation phase (0–5 s). The first bin elicited the largest and most widespread cluster set. Besides the obligatory peri-Rolandic activation, strong foci emerged in the supplementary motor cortex, left superior frontal gyrus, and dorsal striatum (caudate/putamen), consistent with the need to trigger a well-learned motor pattern and release it from basal-ganglia gating. Direct comparison “segment 1 > segment 2” confirmed selective surges in these regions (left SMA/pre-SMA peak t = 6.1; caudate t = 5.4). The second is steady-state phase (5–15 s). Activation then stabilised into a sensorimotor–cerebellar core: the bilateral pre-/post-central gyri, ventral insula, anterior cerebellar hemispheres, and pontine nuclei sustained moderate but constant BOLD levels, whereas frontal–striatal hotspots subsided. No significant differences were detected between segments 2 and 3, indicating a plateau once rhythm and force were established. The third is the termination/re-adjustment phase (15–25 s). In the fourth and fifth bins, cluster volume expanded again but with a shifted centre of gravity. Relative to segment 3, the contrast “segment 4 > segment 3” revealed heightened contralateral cerebellar activity (lobule VI), posterior cingulate and inferior parietal cortex, suggesting on-the-fly updating of jaw kinematics, and sensory re-afference as the bolus consistency changes. A small right inferior frontal/insula cluster resurfaced, possibly reflecting re-engagement of executive monitoring in preparation for the upcoming rest block. Throughout all bins, a compact mid-brainstem cluster—overlapping the putative trigeminal pattern-generator zone—remained tonically active, supporting the idea that cortical and cerebellar nodes modulate rather than generate the basic rhythm [31].

Although no other trial sampled at this granularity, converging evidence aligns with the triphasic scheme. Group-ICA showed that the cognitive–emotional component (IC 2) lags the sensorimotor IC 1 by ~3 s, implying early motor drive followed by executive appraisal [39]. Likewise, the stress paradigm detected a rapid insula/dACC coupling surge in the first seconds of a noise burst that was then curtailed by ongoing chewing [34].

Together, these results indicate that mastication is front-loaded with cortical–striatal command signals, maintained by a sensorimotor cerebellar loop, and fine-tuned again toward block end—a temporal choreography that mirrors the behavioural arc of bite initiation, rhythmic grinding, and bolus assessment before swallowing.

#### 3.1.4. fMRI—Demographic Factors

The fMRI studies of gum chewing used small sample sizes. While participants of both sexes were included, none conducted sex-specific analyses to observe potential differences in brain activation. Therefore, it is not currently possible to definitively link sex as a demographic factor to specific changes in brain activity within this literature. The parameter of participants’ age and its effect on brain activation was described in an earlier section.

Another parameter that has been investigated is handedness. For instance, in study [33], the comparison of the voxel activation of Brodmann’s areas six and four in the right and left hemispheres revealed, for both chewing tasks, a prevalent activation of the right hemisphere in right-handed individuals and a corresponding prevalent activation of the left hemisphere in left-handed individuals.

#### 3.1.5. Summary of Quantitative Effects

Pooling the results of all fifteen fMRI investigations yields a coherent quantitative portrait of human mastication. Across studies, the peri-Rolandic cortex, anterior cerebellum, and thalamus were activated without exception, while the supplementary motor area and premotor cortex appeared in roughly nine out of ten experiments, the posterior insula in about four out of five, the basal ganglia in three-quarters, and limbic or prefrontal foci in just over half. In young adults scanned at 3 T, the mean block-averaged BOLD gain settled near 2% in MI/SI (range 1.6–2.7%), 1.8% in the SMA, and 2.3% in the anterior cerebellum, with cortical peak t-values clustering between 12 and 17 and sub-cortical peaks between 6 and 10.

Mechanical load modulated these amplitudes decisively: replacing a moderately hard gum with a very hard one reduced cortical signals by roughly one-fifth but raised cerebellar responses by one-third [26], and a soft-versus-hard bolus comparison reproduced a 17% voxel-count advantage for the softer stimulus [33]. When chewing was combined with 1 Hz finger flexion–extension, the contralateral SM1 voxel count fell from 2090 to 1296 (−38%), with an even sharper two-thirds drop in S1 alone [28]. Yet, chewing accelerated performance in the Attentional Network Test by 36 ms (−6.4%) without diminishing accuracy and did so in tandem with selective up-regulation of premotor and cingulo-frontal nodes [35].

Affective context exerted an equally measurable influence: during 90 dB noise, the act of chewing lowered mean self-rated stress by one-fifth and simultaneously reversed the direction and halved the strength of STS → anterior-insula drive, thereby dampening insula–dACC coupling [34]. Hemispheric balance proved plastic rather than hard-wired; contralateral dominance tracked the subject’s habitual chewing side [27], disappeared under forced unilateral chewing in right-handers where the lateralisation index never exceeded 0.055 [30], and flipped wholesale with handedness in an unconstrained paradigm [33].

Long-term experience also mattered. Frequent mint-gum users lateralised trigeminal menthol to the correct nostril 21% more often and displayed stronger mid-cingulate and SMA activation than infrequent users, with chewing frequency correlating positively with performance (r = 0.64) [36]. A single hour of unilateral mastication boosted ipsilateral trigeminal principal-nucleus perfusion by 30%, the change scaling with individual side-preference scores [40].

Finally, age reshaped the network dramatically: in middle-aged participants chewing-evoked BOLD in MI/SI, the cerebellum and thalamus fell to about two-thirds of youthful levels, and in the elderly to roughly one-third, whereas right-prefrontal activation rose to 1.7-fold and 4-fold the young-adult baseline, respectively [38]. Taken together, these figures show that, while a bilateral sensorimotor–cerebellar core is obligatory and robust at around a 2% BOLD amplitude, its expression can be attenuated or amplified by 20–40% through instantaneous factors such as bite force, dual-tasking, emotional load, or habitual exposure, and can shift several-fold across the life span—underscoring the striking plasticity of the human masticatory network.

#### 3.1.6. fNIRS Studies

The 12 included fNIRS studies [41,42,43,44,45,46,47,48,49,50,51,52] are presented in Table 2. The fNIRS investigations explored how gum chewing impacts cortical hemodynamics, particularly in the prefrontal cortex (PFC), under varying sensory, cognitive, and emotional conditions. Despite varying focuses—such as flavour, chewing frequency, or stress exposure—several consistent patterns emerged.

##### Participants Characteristics

Across the 12 experiments reviewed, a total of 204 healthy adults took part. Sex was reported for 196 (108 men, 88 women); in one study, the eight volunteers did not disclose sex [44]. All samples comprised neurologically and psychiatrically intact adults, predominately in their 20s (overall mean ≈ 26 years, range 20–37 years) and largely right-handed [41,42,43,44,45,46,47,48,49,50,51,52]. Inclusion criteria were homogeneous: natural dentition, normal (or corrected-to-normal) vision, and no contraindications to near-infrared spectroscopy or Doppler monitoring.

##### Chewing Paradigms Employed Across the fNIRS Literature

The twenty-first-century wave of chewing research that adopted functional near-infrared spectroscopy shares a common methodological foundation: continuous optical monitoring of the frontal cortex during jaw movement. However, the behavioural tasks designed to elicit chewing vary significantly depending on the specific physiological or psychological question being asked. A close reading of the protocols reveals four recurrent paradigm themes.

Two studies emphasised the role of flavour and odour. In one, volunteers chewed either a palatable lemon gum or an aversive salty-liquorice gum for 5 min each, rating pleasantness immediately afterwards while a 55-channel cap tracked haemodynamics over frontal and parietal cortices [41]. A related experiment utilised three gums matched for hardness but differing in taste (sweet vs. neutral) and odour (lemon vs. none); participants chewed blindfolded at 1 Hz for 5 min with ≥5 min wash-out periods [42]. In both designs, motor output was held constant by metronome pacing and comparable masseter EMG, allowing the investigators to attribute observed increases in left-prefrontal oxygenation to sensory appraisal rather than physical effort.

One study combining transcranial-Doppler and fNIRS explored whether chewing laterality or tempo influenced global flow dynamics. Participants completed 5 min bouts of free chewing, right-side chewing, and right-side chewing paced at 1 Hz, each bracketed by 5 min of rest [43]. A complementary NIRS paper varied frequency—30, 70, or 110 chews min^−1^ for three minutes—and found that faster rhythms increased left-PFC oxygenation, even in the absence of taste stimulation [48]. A replication incorporating the same frequencies between two arithmetic tasks confirmed the perfusion effect but did not detect any cognitive enhancement [49].

A cluster of investigations embedded gum chewing within emotionally or cognitively loaded contexts. Two small but carefully controlled experiments paired 30 s blocks of unpleasant International Affective Digitised Sounds (IADS) with either passive listening or concurrent chewing. Utilising a dual-distance HOT-1000 headset, these studies demonstrated that mastication increased total haemoglobin in the right-PFC, raised alpha-EEG power, and made the sounds feel less aversive [46,47]. A parallel line of work coupled chewing with a computerised Stroop test [45] or with treadmill walking while listening to pleasant sounds [50]. In each case, chewing was continuous (60 s in the walking study; 90 s across three Stroop blocks) and self-paced, and fNIRS recorded a selective rise in dorsolateral or ventromedial PFC oxygenation together with modest improvements in reaction time or mood ratings.

Finally, two protocols extended mastication durations beyond the brief optical-measurement periods to investigate systemic physiological effects. One team involved participants performing 20 min of rhythmic chewing whilst a 24-channel array sampled the whole PFC. Immediately afterwards, researchers quantified blood serotonin and nociceptive-flexion reflexes. This investigation revealed a ventrolateral-PFC-centred oxygenation rise that paralleled a 7% jump in 5-HT and a sustained drop in pain reflex amplitude [51]. The other group implemented a 30 s chew/30 s rest block design but deliberately displaced the optode over the temporal muscle in half the runs; the resulting surge in “cortical” signal under the deviated probe demonstrated that optode positioning is as crucial as the masticatory task itself [44].

##### Cortical Hemodynamic Responses to Mastication

Chewing gum, irrespective of flavour or experimental context, triggers a rapid and reliable rise in cortical oxygenation. Every study using fNIRS or broadband NIRS recorded significant increases in oxygenated haemoglobin (Δ[oxy-Hb]) or total haemoglobin (Δ[total-Hb]) over bilateral sensorimotor cortices and, to varying extents, the prefrontal cortex within 5–10 s of jaw movement, with a plateau 20–40 s later. Complementary Doppler work showed middle-cerebral-artery velocity climbing 15–18% above baseline and peaking at 114–148 s, again independent of motor constraints or flavour content [42,43]. Sensory valence clearly modulated prefrontal recruitment: an unpalatable salty-liquorice gum elicited a markedly larger left-lateralised frontopolar/dorsolateral prefrontal response than a palatable lemon gum (peak Δ[oxy-Hb] ≈ +0.45 µM vs. +0.20 µM; area-under-the-curve +27%, *p* < 0.01) [41], while adding lemon odour to a sweet gum almost doubled the bilateral frontal signal relative to taste-only gum, with the effect emerging after ~120 s, peaking at ~180 s and persisting two minutes into recovery (flavour × time F = 6.11, *p* = 0.004) [42].

Motor parameters exerted subtler influence. Forcing participants to chew solely on the right or to match a 1 Hz metronome, left cerebrovascular responses, heart rate, and masseter EMG unchanged relative to free chewing, indicating that cortical drive is robust against moderate side or rhythm manipulations [43]. In contrast, chewing speed produced a clear dose effect: in two NIRO-200 studies, fast mastication (≈110 chews min^−1^) generated 2–3-fold larger left-PFC Δ[oxy-Hb] than normal (70 cpm) or slow (30 cpm) rates (linear trend *p* ≤ 0.001) and was the only frequency that heightened PFC oxygenation during a subsequent arithmetic task [48,49].

Context further shaped the response. When pleasant sounds accompanied the task, both chewing alone and chewing while walking boosted ventromedial/orbitofrontal Δ[total-Hb] by ~0.12 mM·mm versus rest, whereas walking without gum showed only a non-significant trend [50]. Under aversive IADS sounds, chewing quintupled right-PFC haemodynamics (0.120 mM·mm vs. 0.027 mM·mm, *p* = 0.0006) and simultaneously increased alpha-wave appearance and subjective comfort [46]; a denser 16-channel array confirmed a 90% larger oxy-Hb rise in medial right PFC when chewing accompanied listening (*p* < 0.01) [47]. Duration also mattered: a 20 min rhythmic bout produced a sustained ventrolateral/ventromedial activation (mean z-score +3.79 ± 1.04) while dorsal PFC returned to baseline (−0.08 ± 1.00), mirroring a concurrent 7.7% serotonin surge and nociceptive-reflex suppression [51].

Because chewing activates temporal and facial muscles, artefact control is critical. Dual-distance recordings showed that, with the probe centred over the PFC, chewing elevated deep-cortical and motion-corrected “neural” signals but not the 1 cm superficial channel; lateral displacement onto the anterior temporal muscle abolished this dissociation, with all channels rising equally and obscuring neural activity [44].

Finally, haemodynamic amplification did not always translate into behavioural gain. Although chewing accelerated Stroop responses alongside heightened left-DLPFC oxygenation [45], it failed to improve serial-recall accuracy or Uchida–Kraepelin arithmetic scores despite transient PFC hyper-oxygenation [49,52], emphasising that increased perfusion alone is insufficient for cognitive enhancement and that task domain, difficulty, and baseline engagement modulate any functional benefit. Collectively, these findings depict a robust mastication-evoked haemodynamic signature—sensorimotor activation accompanied by frequency-, flavour-, and context-dependent recruitment of ventrolateral and ventromedial prefrontal networks involved in emotion and executive control.

##### Peripheral Physiological Correlates

- Cardiovascular dynamics

Chewing invariably provoked a modest tachycardia. In the 5 min flavour-valence study, heart rate (HR) rose within 10 s of chewing onset and remained ~7% above baseline until the first post-chew minute, with no HR difference between palatable and unpalatable gums [41]. Under side- and rhythm-controlled chewing HR peaked at 105–120 s (grand-mean Δ ≈ +10 bpm), yet was statistically indistinguishable across the three motor conditions (F_2,48_ = 0.71, *p* = 0.50) [43]. When negative IADS sounds were paired with mastication, HR climbed from 73.65 ± 6.0 bpm (listen only) to 77.69 ± 6.3 bpm (listen + gum), t_11_ = 3.00, *p* = 0.010 [46]; an independent sample yielded a comparable shift (61.91 ± 5.4 → 67.83 ± 5.7 bpm, *p* = 0.017) [47]. Pleasant-sound chewing, walking, or their combination produced parallel 5–8 bpm accelerations without between-task differences [50].

- Autonomic balance

Spectral ECG analysis revealed no flavour-dependent change in high-frequency power (HF) or LF/HF ratio during chewing (both *p* > 0.20) [41], suggesting parasympathetic–sympathetic balance is preserved. Likewise, rhythmic versus free chewing left LF/HF unchanged (F_2,48_ = 0.64, *p* = 0.53) [43].

- Cranio-facial muscle activity

Masseter surface-EMG confirmed equivalent motor effort across most paradigms. Lemon and salty-liquorice gums elicited identical integrated EMG envelopes (ΔEMG ≈ 45% MVC each) [41]. Free chewing produced symmetric left/right activity, whereas forced right-side chewing boosted right-masseter output by +18% MVC and lowered chewing frequency from 75.5 ± 6.9 to 60.4 ± 4.8 cpm (both *p* < 0.01) without altering cerebral perfusion [43].

- Cerebrovascular correlates

Transcranial Doppler showed that middle-cerebral-artery velocity (MCAV) increased 15–18% above resting flow in all three chewing modes, peaking 114–148 s after onset; amplitudes did not differ by side or rhythm (interaction F < 1.0) [43]. These Doppler findings dovetail with the fNIRS oxy-Hb rises reported in Section 4.2, underscoring tight neurovascular coupling during mastication.

- Neurochemical and nociceptive markers

A single 20 min rhythmic bout elevated whole-blood serotonin by 7.7 ± 1.1% immediately post-chew and maintained this elevation 30 min later (ANOVA F_2,27_ = 12.7, *p* < 0.001) [51]. Concomitantly, the nociceptive flexion-reflex (NFR) area fell 24% relative to baseline (F_2,27_ = 16.2, *p* < 0.001), linking serotonergic up-regulation, ventral-PFC activation, and analgesia.

- Central–autonomic integration

EEG alpha-wave appearance—a marker of relaxed alertness—increased during chewing under auditory stress (44.43 ± 3.9% vs. 42.78 ± 3.5%, *p* = 0.028) [46] and was replicated with a denser montage (47.10 ± 4.2% vs. 44.00 ± 3.8%, *p* = 0.031) [47]. These oscillatory shifts ran parallel to right-PFC haemodynamic amplification, suggesting an integrated cortico-autonomic response that supports affect regulation.

Taken together, peripheral indices confirm that the haemodynamic activation is accompanied by sympathetic-leaning yet physiologically moderate cardiovascular arousal, stable autonomic balance, muscle work proportional to chewing load, and—in the case of prolonged rhythmic chewing—biochemically mediated hypoalgesia.

##### Behavioural, Cognitive, and Affective Outcomes

- Palatability effects

Flavour pleasantness scales inversely with left-prefrontal load: aversive salty-liquorice evokes the greatest frontopolar/DLPFC activation, whereas multisensory enrichment (sweet + lemon odour) maximises both perceived flavour and bilateral frontal oxygenation [41,42].

- Stress attenuation

During low-pleasure IADS exposure, mastication consistently improves affect ratings by 8–15 VAS points, elevates right-ventromedial PFC total-Hb five-fold and increases alpha-EEG activity, denoting an integrated cortico-autonomic coping response [46,47].

- Cognitive performance

Chewing accelerates tasks requiring inhibition/attentional switching (Stroop) [45] but leaves rote serial recall [52] and sustained arithmetic [49] unchanged, highlighting domain-specific benefits.

- Frequency and dual-task factors

High-cadence mastication (110 cpm) prolongs PFC hyper-oxygenation yet still fails to enhance arithmetic output [48,49]. Combining gum with low-intensity walking does not add cortical or affective gain beyond chewing alone, suggesting a ceiling effect under light workloads [50].

- Analgesic outcome

Prolonged rhythmic chewing activates ventral PFC, elevates circulating serotonin, and reduces the nociceptive flexor reflex for ≥30 min, consistent with recruitment of a serotonergic descending pain-inhibitory pathway [51].

In sum, gum chewing reliably boosts positive affect under emotional challenge, speeds selected executive processes, and confers short-term hypoalgesia, whereas memory or arithmetic gains are absent—effects that depend on flavour valence, chewing cadence, and task context.

##### Methodological Considerations for fNIRS Chewing Gum Studies

Across the twelve experiments, eight different fNIRS/NIRS instruments were employed, yet all converged on a broadly comparable optical geometry: continuous-wave light at three wavelengths clustered near 780, 805, and 830 nm; emitter–detector separations of 30–40 mm to sample cortex at ~15–25 mm depth; and sampling rates between 1 Hz and 10 Hz. The multichannel LABNIRS (55 channels, 3.3 Hz) mapped frontal–parietal activation during flavour valence manipulation [41], while the 24-channel OMM-3000 (130 ms raw rate) provided voxel-registered ventral–dorsal prefrontal contrasts for prolonged chewing [51]. Compact two-channel, dual-distance devices (HOT-1000 and HOT-2000) paired a 1 cm “superficial” detector with a 3 cm “deep” detector, enabling real-time subtraction of scalp and muscle signals—a critical safeguard when jaw and temporal muscles are active [44,46,50]. Conventional single-distance systems without short-separation channels (e.g., NIRO-200, NIRO-300, OM-100) relied instead on meticulous optode placement clear of the anterior temporal muscle, low-pass filtering (0.1–0.2 Hz), and five- to ten-point moving averages to attenuate motion noise [42,48,52].

Probe placement followed the 10–20 EEG grid in every study, but subtle deviations proved decisive. When the HOT-1000 probe was centred at Fp1/Fp2, chewing raised deep-and-“neural” total-Hb while the superficial channel remained flat, confirming cortical origin. Shifting the probe 20 mm laterally onto the anterior temporal muscle caused all three channels—including the motion-corrected one—to rise in parallel, obliterating neural specificity (superficial > deep; *p* < 0.001) [44]. This single manipulation illustrates why short-separation correction alone is insufficient without anatomically faithful optode placement.

Baseline definition and block timing were broadly standardised: at least 20–30 s of seated rest before each chewing block and an equivalent post-task rest ensured that slow systemic drifts were removed with linear baseline correction [41,42,43,44,45,46,47,50,51,52]. Haemodynamic modality separation (tissue vs. systemic oscillations) was additionally performed in the LABNIRS study via the NIRS-SPM “HMS” routine, which helped isolate cortical signals from skin blood flow and respiration components [41].

Anatomical localisation ranged from single-shot scalp coordinates (Fpz, Fp1/Fp2, or 2 cm lateral to midline) [48,49,50] to full 3-D digitisation with MRI co-registration and MNI channel labelling [41,51]. Where digitisation was used, regions-of-interest (frontopolar = BA 10; DLPFC = BA 9/46; ventromedial/orbitofrontal = BA 11/47) could be linked to behavioural phenomena (e.g., valence, analgesia) with millimetre precision.

Motion artefact control involved (i) rigid plastic or elastic caps, (ii) chew-side standardisation or metronome pacing in five studies [41,42,43,48,49], and (iii) on-board accelerometers in the HOT series instruments [44,46,50]. Despite these measures, low-frequency “chewing harmonics” (<2 Hz) can alias into the haemodynamic band if the sampling rate is <4 Hz—a risk recognised and avoided in all but one 1 Hz study, which therefore restricted analysis to 1 min means rather than finer temporal windows [42].

Complementary peripheral measures—masseter EMG in five studies [41,43,45,51,52], heart rate optical sensors in four [46,47,50,51], and transcranial Doppler in one [43]—verified that cortical oxygenation changes could not be attributed to differences in chewing force, cardiac output, or global CBF. Notably, MCAV rose uniformly across rhythm-controlled and free-chew conditions, reinforcing the validity of fNIRS findings even when focal cortical topographies differed [43].

Taken together, the mastication literature demonstrates that reliable cortical recordings require three non-negotiables: (1) an emitter–detector spacing of ≥30 mm to reach cortex; (2) either a co-located short-separation channel or meticulous avoidance of facial muscles; and (3) baseline-anchored block designs long enough (≥20 s rest, ≥30 s chew) to permit filtering below 0.1 Hz. When these criteria are met, chewing-related haemodynamics can be captured with signal-to-noise ratios comparable to seated cognitive paradigms, enabling confident interpretation of flavour, frequency, and affect manipulations across studies [41,42,43,44,45,46,47,48,49,50,51,52].

##### fNIRS—Demographic Factors

Studies employing fNIRS included participants of both sexes in approximately equal proportions; however, no study analysed sex-specific groups separately. Furthermore, these studies did not examine the influence of participants’ age or handedness on fNIRS measurements.

##### Synthesis

Taken together, the twelve studies reviewed portray mastication as a multimodal neural–behavioural driver that recruits a stereotyped sensorimotor core and a context-sensitive prefrontal shell. fNIRS consistently captured an initial increase in oxy-Hb over the peri-rolandic chewing area within seconds of jaw movement, a response that was impervious to side-of-bite or rhythmic constraint and paralleled a 15–18% rise in middle-cerebral-artery velocity [42,43]. Superimposed on this motor backbone, the prefrontal cortex (PFC) reacted in a graded, domain-specific manner: left-lateralised DLPFC/frontopolar activity grew when chewing was aversive [41], bilateral frontal oxygenation doubled when odour enriched flavour salience [42], ventromedial/orbitofrontal signals climbed during pleasant-sound chewing [50], and right-ventromedial PFC responses were amplified when gum buffered aversive sounds, tracking improved affect and alpha-EEG indices of relaxed alertness [46,47]. Faster mandibular cadence (≈110 cpm) augmented and prolonged left-PFC oxygenation beyond the chewing epoch [48,49], whereas a 20 min bout selectively sustained ventrolateral/ventromedial—but not dorsal—PFC activation and coincided with a 7–8% systemic serotonin surge and a 24% suppression of the nociceptive-flexor reflex [51].

Behaviourally, these haemodynamic signatures translated into selective functional gains. Chewing accelerated Stroop performance (≈−50 ms) when executive inhibition was required [45], mitigated discomfort during affective challenge by 8–15 VAS points [46,47], and induced short-lived hypoalgesia [51]. In contrast, rote serial recall and sustained addition were resistant to either transient or cadence-manipulated PFC hyper-oxygenation [49,52], underscoring that perfusion up-regulation alone is insufficient for cognitive enhancement and that task domain, baseline load, and stimulus salience govern any benefit.

Peripheral physiology corroborated the cortical picture but remained modest in magnitude. Heart rate acceleration was limited to 4–10 bpm, showed no flavour or rhythm specificity, and kept LF/HF ratios unchanged [41,43,46,47,50]. Masseter EMG confirmed equivalent chewing effort across flavour and dual-task conditions [41,43], and Doppler flow increases paralleled but did not dictate the topography of PFC change [43]. Thus, the prefrontal effects cannot be reduced to generic cardiovascular arousal or muscle load.

Methodologically, the corpus demonstrates that reliable mastication neuroimaging hinges on three safeguards: a ≥30 mm source–detector span to reach cortex, short-separation (1 cm) channels or scrupulous avoidance of the temporal muscle, and baseline-anchored blocks long enough to filter <0.1 Hz drifts [41,42,43,44,50]. When these criteria were met, signal-to-noise ratios matched those of seated cognitive paradigms, and ventral–dorsal PFC dissociations could be resolved with millimetre accuracy after MRI-assisted channel mapping [41,51].

In sum, chewing is a potent, tunable activator of prefrontal circuitry whose magnitude is scaled by flavour valence, multisensory enrichment, chewing cadence, emotional context, and task duration. The convergent evidence supports a mechanistic model in which sensorimotor drive gates afferent taste–odour and emotional inputs into ventrolateral/ventromedial PFC, engaging autonomic and serotonergic pathways that modulate affect and pain while selectively sharpening executive speed. Limitations include small, young, and healthy samples, diverse cognitive tasks, and short-term designs. Future work should couple fNIRS with fMRI or high-density EEG, extend sampling to older or clinical cohorts, and test sustained mastication interventions to clarify whether the observed cortical and behavioural effects can translate into durable cognitive or emotional benefits.

#### 3.1.7. EEG Studies

The seven included EEG studies [46,47,53,54,55,56,57] are presented in Table 3. These EEG-based studies reinforce the notion that gum chewing meaningfully alters brain activity, though the specific patterns of change depend on factors such as flavour, sugar content, the presence of aromatic oils, and the timing of measurement (i.e., during or after chewing). While the precise mechanistic pathways are still under investigation, several key themes emerge.

##### Chewing Paradigms Employed Across the EEG Literature

Across the EEG literature, chewing protocols vary considerably, with each method eliciting distinct electromyographic artefacts detectable at the scalp level. In the sustained-attention experiment [53], participants completed four consecutive 5 min 30 s digit-monitoring blocks; tasteless, medium-hard gum was chewed only during the second block, then discarded. EEG was recorded exclusively in eyes-closed resting intervals prior to chewing, immediately after chewing, and ten minutes later, ensuring muscle artefacts did not contaminate the recordings [53].

A series of Japanese “flavour-dissection” studies separated motor rhythm from chemosensory input. In the study by Masumoto et al., participants chewed for one minute on a bolus containing either gumbase alone, gumbase + sucrose, or gumbase + spearmint oil (plus, on separate days, odour inhalation without chewing): EEG recordings were confined to pre- and post-rest periods [54]. Morinushi et al. employed the same 1 min rhythm but contrasted a tasteless base with an otherwise identical gum laced with aromatic oil [56]. Yagyu et al. extended chewing to two minutes and contrasted a multi-flavoured commercial “Relax Gum” with plain gumbase [57]. In all cases, EEG data was sampled before and after mastication, allowing flavour-specific changes—typically in the α- and β-band shifts—to be observed without jaw-muscle noise [54,56,57].

A third experimental paradigm embeds chewing into an aversive-auditory challenge. In two companion studies [46,47], participants were exposed to six 30 s blocks of unpleasant IADS alternating with brown-noise rests. In one condition, a tasteless 1 g gum was chewed continuously throughout all three minutes of stimulation. As the outcome measure was alpha-wave appearance rate (an index of relaxed alertness), EEG was recorded during epochs during active chewing. Modern artefact-correction routines were applied to extract interpretable spectral data.

Other researchers employed longer rhythmic chewing as a physiological manipulation. Masumoto et al. placed a three-minute spearmint-gum bout between two five-minute resting recordings and observed an immediate, transient upward shift in alpha frequency, a marker of heightened arousal [55].

Despite these differences, several consistent methodological features emerge. Brief sensory-oriented chewing protocols are paced at approximately 70 strokes min^−1^ and are bracketed by artefact-free recordings [54,56,57]. Stress interaction protocol recorded EEG data continuously and relied on signal-cleaning, as the chewing–stimulus interplay itself constituted the phenomenon of interest [46,47]. Furthermore, prolonged chewing paradigms suspend EEG data acquisition until the jaw was still, thereby allowing after-effects on arousal to be captured without muscle contamination [55].

##### Participant Characteristics

Across the seven studies reviewed [46,47,53,54,55,56,57], a total of 123 healthy adults were examined. Mean ages clustered in the mid-twenties (overall range 24–34 years). All volunteers were free of major medical, neurological, or psychiatric conditions, and none were regular gum chewers. Handedness was reported in two studies (right-handed only). Sampling was deliberately homogeneous to minimise inter-individual variability in resting EEG measures, but the proportion of women varied markedly (8–45%). Sample sizes were modest (*n* = 9–40), restricting the power of single studies yet enabling detailed within-subject analyses.

##### Electroencephalographic Outcomes

Across all seven studies, chewing gum produced a characteristic but composition- and context-dependent modulation of cortical rhythms. The clearest and most consistent signature was a transient elevation of beta power (13–30 Hz), first demonstrated in the large vigilance experiment [53] where frontal and temporal beta rose immediately after mastication, tracking the short-lived heart-rate surge and faster reaction times. Similar beta enhancement re-appeared whenever participants chewed plain gum base in resting protocols [54] at multiple fronto-parietal leads and when flavour or aroma was added [56,57], although the topography shifted anteriorly and inferiorly for sensory-rich gums, suggesting recruitment of additional prefrontal networks. By contrast, adding sucrose without flavour dampened beta activity and heightened theta, hinting at a mildly sedative metabolic effect [54].

Alpha activity (8–13 Hz) displayed a complementary but more nuanced pattern. During the vigilance task, it remained flat despite behavioural gains, implying that the arousal benefit was driven chiefly by beta-linked mechanisms. In quiet rest, however, chewing tasteless gum consistently amplified alpha: mean alpha frequency crept upward in nine of twelve scalp sites [55], global alpha power increased over posterior and midline regions while beta fell [56], and source modelling revealed a posterior shift of alpha-2 generators coupled with subjective feelings of comfort [57]. When flavour and aroma were present, alpha still rose but was now accompanied by focal beta gains and widespread theta suppression, a constellation the authors of [56] labelled “relaxed concentration”—heightened external attentiveness without loss of calm. This dual profile also emerged under emotional stress: in two independent fNIRS–EEG studies [46,47], unpleasant sounds suppressed alpha, yet concurrent chewing restored the alpha appearance rate and nudged beta upward, signifying a cushioning of stress reactivity.

In the lower bands, theta (3–8 Hz) generally declined whenever beta climbed—particularly with flavoured gums or intense aroma exposure—reinforcing the interpretation of diminished drowsiness and greater outward focus. Delta was seldom analysed and showed no reproducible change. Taken together, the EEG data portray mastication as a phasic cortical activator whose mechanical component briefly boosts beta-mediated alertness, while gustatory and olfactory inputs broaden the response into a balanced state of high engagement and calm, and stressful environments reveal an additional protective, alpha-restorative effect.

##### Behavioural and Autonomic Correlates

Chewing gum consistently produced fast-acting, yet predominantly short-lived, changes in behaviour and peripheral physiology that mirrored the EEG findings. In the largest experiment [53], mastication accelerated simple vigilance: reaction times fell by ~25 ms (*p* ≈ 0.05) and correct detections rose during the chewing block, only to subside to baseline within one post-chewing session—precisely the time window in which frontal/temporal beta power and heart rate were elevated. Heart-rate acceleration itself was striking (≈ +6–8 beats min^−1^, *p* < 0.001) but disappeared once chewing stopped, underscoring the phasic nature of the arousal. In the two stress-challenge studies [46,47] unpleasant sounds depressed alpha activity and increased self-reported discomfort, yet simultaneous chewing partially normalised the alpha wave appearance rate and reduced subjective distress, with medium-to-large effect sizes (Cohen’s d ≈ 0.8). These neurophysiological cushions were accompanied by modest dampening of heart rate increases and prefrontal haemodynamic responses, suggesting that rhythmic mastication blunts emotional reactivity at both cortical and autonomic levels. Flavour further shifted subjective state: participants chewing sensory-rich gum felt more “refreshed” and “comfortable” (*p* ≤ 0.001) than after tasteless gum [57], dovetailing with the mixed alpha-plus-beta EEG profile that typifies relaxed alertness. Conversely, adding sucrose alone did not enhance mood or vigilance and even produced a theta-dominant EEG pattern viewed as mildly sedative [54]. No study reported adverse mood effects, indicating that gum chewing—particularly when flavoured—tends to either boost alertness or buffer stress without precipitating negative affect. Taken together, behavioural gains, cardiovascular spikes, and favourable subjective shifts all converge on the notion that mastication acts as a rapid, context-sensitive modulator of arousal, the magnitude and valence of which are tuned by flavour complexity and situational demand.

##### EEG—Demographic Factors

EEG studies involving gum chewing have typically included small sample sizes, with participants of both sexes. No division of participants into groups by age, handedness, or sex was undertaken in these investigations. Consequently, none of the studies measured differences in EEG brain activity according to these parameters.

## 4. Discussion

Chewing gum is a very common, seemingly mundane activity. In the world of neuroscience, numerous works have thoroughly explored the effects of this activity on various states of human functioning. In this mechanistic review, we examined the neural effects of gum chewing, searching for studies that used different neuroimaging techniques. These experiments allowed us to capture these changes. Gum chewing appears to be an activity that induces a cascade of functional, haemodynamic, and bioelectrical changes. This may directly and indirectly explain why gum chewing improves cognitive functions, reduces stress, etc.

### 4.1. fMRI Outcomes

**Figure 2:**
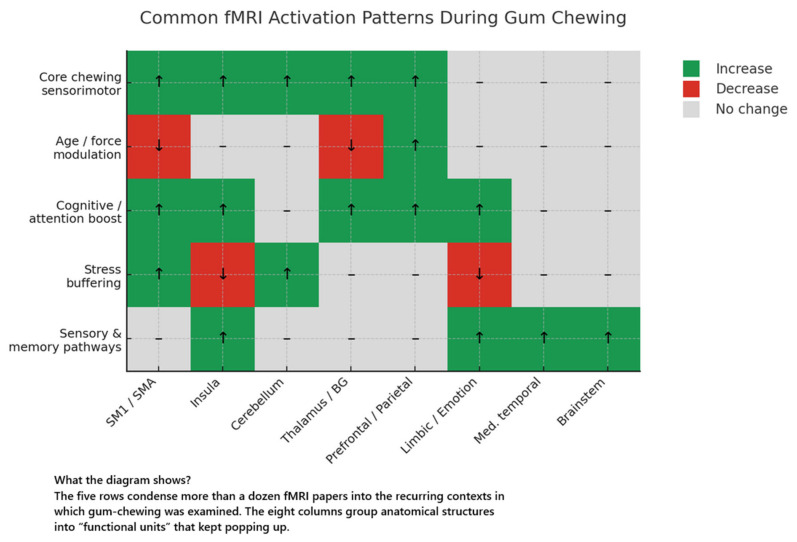
Common fMRI activation patterns during gum chewing.

Collectively, the fMRI studies reviewed here provide a detailed picture of how chewing gum engages widespread neural networks that extend well beyond the primary sensorimotor areas responsible for basic jaw movement. Across multiple experiments using block designs, rhythmic chewing paradigms, and comparisons of gum hardness and flavour, a consistent finding is robust bilateral activation in the primary motor (M1) and somatosensory (S1) cortices, supplementary motor area (SMA), insula, thalamus, and cerebellum. These regions are known to coordinate mastication and integrate somatosensory feedback [58,59,60,61,62], underscoring chewing as a complex motor behaviour requiring precise coordination of jaw muscles and oral structures. A description of the common findings based on fMRI is presented in Figure 2.

#### 4.1.1. Sensorimotor Activation and Modulatory Variables

A prominent and consistent finding across multiple fMRI studies is robust activation of the sensorimotor system during gum chewing. This system includes the primary motor cortex (M1; BA4), primary somatosensory cortex (S1; BA3, BA1, BA2), supplementary motor area (SMA; BA6), and adjacent sensorimotor-associated regions such as the premotor cortex, insula, and cerebellum. Notably, these activations occur bilaterally—even when chewing unilaterally—and can be modulated by variables such as gum hardness, chewing-side preference, and the temporal phase of the chewing sequence.

Several studies demonstrate that unilateral chewing triggers symmetrical BOLD signals across both hemispheres, suggesting that the need to stabilise the jaw and maintain rhythmicity requires bilateral control. One representative example is study [30], where lateralisation indices for unilateral chewing were extremely low (<0.055), indicating near-equal representation in both hemispheres. Similarly, research on chewing different gum hardness levels [26] indicates that both hard and moderately hard gum activate the M1, S1, SMA, insula, thalamus, and cerebellum. However, moderately hard gum yielded stronger cortical responses (sensorimotor cortex, SMA, and insula), whereas harder gum triggered greater cerebellar engagement. This suggests that increased masticatory effort shifts peak activity from cortical to cerebellar regions. Chewing-side preference (CSP) further influences these patterns: Study [27] found that left- or right-sided CSP exhibited contralateral dominance of sensorimotor activation, suggesting that habitual unilateral chewing can bias cortical responses toward the preferred side.

Beyond such spatial patterns, researchers have explored the temporal dynamics of chewing. In study [31], a block-design approach segmented chewing periods into shorter intervals, revealing that early phases preferentially engage higher-order motor planning structures (e.g., the putamen and caudate nucleus), while later phases highlight cerebellar and temporal areas involved in motor coordination and timing. Similarly, study [37], which compared spontaneous chewing to slow, deliberate jaw movements, likewise observed a stronger cerebellar signal during rhythmic, internally guided chewing. This highlights the cerebellum’s role in automating repetitive motor patterns.

Collectively, these findings confirm that chewing gum extends beyond a simple jaw reflex. Rather, it involves extensive cortical and subcortical circuits regulating motor control, sensory feedback, and continuous real-time adjustments. Variables specific to chewing—such as bolus hardness, side preference, and pacing—can shift the balance of activation between cortical and cerebellar centres, underscoring the flexibility and complexity of this routine oral motor task.

#### 4.1.2. Higher-Order Cortical Regions and Cognitive Correlates

Beyond the primary sensorimotor areas, several fMRI studies demonstrate that gum chewing can engage higher-order cortical regions involved in attention, executive functions, emotional processing, and memory. These findings suggest that mastication has a broader cognitive–emotional impact than mere jaw movement. For example, one study [32] showed that actual gum chewing—compared to a “sham” chewing task—selectively activated the middle frontal gyrus, inferior frontal gyrus, and inferior parietal lobule, regions linked to executive control, working memory, and sensorimotor integration. Such an outcome indicates that the real act of mastication recruits additional cognitive resources beyond simply mimicking chewing movements. Meanwhile, another investigation [35] reported increased activation in the anterior cingulate cortex (ACC) and left frontal gyrus during gum chewing. Although the study did not find significant improvements in specific attentional-network measures, participants showed faster overall reaction times, implying that enhanced prefrontal involvement may facilitate general alertness or response readiness.

Certain studies also connect chewing to stress and emotional processing. In one study [34], gum chewing attenuated activation in the superior temporal sulcus (STS) and anterior insula when participants were exposed to a loud noise. Psychophysiological interaction (PPI) analysis further revealed that chewing disrupted stress-related signal propagation by reducing functional connectivity between the insula and the dorsal anterior cingulate cortex (dACC). These results align with evidence in other work [27,31,33] showing that the insula, which is central to interoceptive awareness and affective modulation [63,64], often responds robustly to chewing. Subjective ratings in some studies also suggest that mastication can momentarily ease stress and heighten comfort or mood. This is supported by evidence from several studies that have shown that chewing gum reduces stress and anxiety levels [17,20,21,65,66,67,68,69]. Different brain circuits are reorganised for processing salient information when exposed to acute stress [70,71]. The anterior insula (AI), a crucial hub of the salience network (SN), is regarded as a principal brain region in the integration of emotions, cognition, physiological states, and interaction with other vital networks such as the central executive network (CEN) and the default mode network (DMN) [72,73,74]. Chewing gum and increasing insula activity may block negative affective signals associated with stress and inhibit excessive interoceptive sensitivity.

In addition, research has begun examining memory-related structures. One study [29] documented significant activation in the left hippocampus and entorhinal cortex (BA28) during chewing, pointing to possible influences on medial temporal lobe (MTL) circuits that underlie memory formation [75,76,77,78,79]. Although these findings alone do not confirm that chewing enhances episodic or working memory, hippocampal engagement hints that masticatory action might, at least transiently, modulate limbic pathways important for learning and recall.

At the same time, chewing can compete with other motor or cognitive tasks for cortical resources. For example, in a study examining hand movements [28], concurrent gum chewing decreased cortical activation associated with finger control, suggesting a redistribution of attentional and motor-processing capacity when two motor tasks must be coordinated. Whether chewing could similarly interfere with higher cognitive tasks remains unclear, as some studies report short-term performance gains in alertness, while others find no robust improvements. Overall, these fMRI findings indicate that gum chewing engages frontal, parietal, and limbic structures beyond basic mastication circuits, potentially contributing to subtle enhancements in alertness, possible stress reduction, and potential —though not yet conclusively demonstrated—memory-related functions.

#### 4.1.3. Medial Temporal Lobe and Memory-Related Activation

While much of the literature on gum chewing focuses on sensorimotor and prefrontal activity, evidence also suggests that masticatory actions engage the medial temporal lobe (MTL)—a region integral to learning and memory [80,81,82,83,84]. A key example comes from study [29], which specifically examined whether gum chewing stimulates MTL structures. Participants performing a chewing task exhibited significant BOLD signal increases in the left hippocampus and entorhinal cortex (BA28), with additional activation in the parahippocampal cortex (BA36). These areas are closely linked to memory encoding and consolidation processes, raising the possibility that chewing could transiently modulate neural pathways involved in memory formation.

Although study [29] did not directly assess memory performance, the finding that gum chewing activates core memory-related regions aligns with broader evidence that sensorimotor activities can influence hippocampal function—often through arousal or interconnections with other limbic areas (e.g., the insula, cingulate cortex) [85]. However, because most studies prioritise immediate neural responses over longer-term cognitive outcomes, it remains unclear whether this MTL engagement translates into observable improvements in memory tasks. Future research that integrates explicit behavioural measures (e.g., episodic or working memory assessments) alongside fMRI could clarify whether chewing-induced hippocampal activation leads to tangible benefits for learning and retention.

#### 4.1.4. Task Interference and Motor Resource Allocation

One key question is whether gum chewing competes with other concurrently performed tasks for motor-related cortical resources. Study [28] directly addressed this by comparing finger flexion–extension movements performed alone versus the alongside chewing. The results revealed that the total voxel count in the contralateral primary sensorimotor cortex (SM1) decreased from 2090 voxels (finger movements alone) to 1296 voxels (finger movements plus chewing). This indicates a substantial reduction in cortical activation dedicated to hand motor control when mastication is added. This contrast suggests that chewing triggers partial resource reallocation or an inhibitory gating mechanism, whereby neural circuits for chewing and limb movement overlap to some degree and thus cannot both operate at maximal capacity at once.

Such interference is consistent with the broader principle that cortical motor networks have limited capacity to control multiple actions simultaneously, particularly if those actions demand precise coordination [86,87,88,89]. Although chewing is typically considered an automatic or subcortical function, the findings underscore that it still recruits higher-level sensorimotor areas in a way that can reduce the cortical “bandwidth” available for other motor tasks. From a practical standpoint, this interference may be subtle in daily life, as routine chewing does not usually coincide with complex motor tasks. Nonetheless, in contexts where fine or demanding manual control is required, concurrent chewing could influence performance by transiently diverting sensorimotor resources. Future research might illuminate whether specific parameters of chewing (e.g., rate, hardness of gum) increase or diminish this interference effect and how the brain dynamically manages competing motor demands.

#### 4.1.5. Dynamics of Chewing and Temporal Segmentation

While many fMRI studies treat chewing as a uniform block condition, some have used temporal segmentation to reveal how neural activation unfolds across successive phases of mastication. Study [31], for example, divided 25 s chewing blocks into five consecutive 5 s segments. By analysing these shorter time frames, the authors observed that distinct brain regions peak at different stages in the chewing cycle. During the initial segment (0–5 s), participants displayed heightened activity in left frontal and subcortical areas—including the superior frontal gyrus, caudate nucleus, and putamen—regions typically associated with motor planning, movement initiation, and habit formation. In the subsequent segments (5–15 s), activation expanded to include premotor and sensorimotor cortices, indicating an interplay of ongoing motor control and somatosensory feedback that supports a stable chewing rhythm. By the final segments (15–25 s), the data showed a pronounced shift toward the cerebellum and temporal lobe structures such as the superior temporal gyrus. Here, the cerebellum likely mediates fine-tuning and error correction for an established chewing cycle, while the temporal region may integrate auditory or other sensory cues that help maintain consistency.

These observations underscore that chewing is not simply a repetitive reflex but a phasic process engaging distinct neural resources over time. Early phases rely on subcortical loops (e.g., caudate and putamen) to initiate the sequence, while later stages recruit the cerebellum for rhythmic coordination. Related findings from other studies [37] comparing spontaneous (habitual) to controlled (deliberate) chewing reinforce this view, showing that neural activation shifts dynamically depending on the automaticity or effort involved.

#### 4.1.6. Unresolved Questions About Central Pattern Generators

A longstanding hypothesis, rooted largely in animal research, posits that rhythmic mastication is driven by a central pattern generator (CPG) located within the brainstem, particularly the trigeminal motor nucleus and reticular formation [90,91,92]. This view is supported by neurophysiological studies in rodents and other mammals, where rhythmic jaw movements can persist even if higher cortical inputs are reduced or absent [93]. However, human fMRI data have not consistently confirmed explicit brainstem activation that would definitively pinpoint such a CPG for chewing. For example, in study [37], both spontaneous and controlled chewing activated extensive cortical and subcortical circuits but showed no clear BOLD signal or connectivity changes within the brainstem, casting doubt on straightforward detection of a CPG via conventional fMRI protocols.

Several methodological and physiological factors might explain this discrepancy. First, the brainstem’s small size and proximity to air-filled cavities can produce susceptibility artifacts in fMRI, limiting spatial resolution and signal quality. This is particularly problematic for detecting subtle fluctuations in smaller nuclei, where even slight head motion or physiological noise can obscure meaningful activity. Second, it remains possible that the brainstem CPG operates at a background level overshadowed by stronger cortical or cerebellar signals in standard whole-brain analyses—especially if participants are asked to perform conscious, goal-directed chewing rather than a purely automatic behaviour. Third, BOLD responses in the brainstem may not follow the same haemodynamic profile as cortical regions, further complicating direct comparisons or threshold-based group analyses.

These methodological constraints do not rule out the existence of a brainstem-based CPG; rather, they underscore the challenges in capturing subtle signals from deep, small, and physiologically noisy structures with standard neuroimaging techniques. Future investigations might employ higher-field MRI (e.g., 7T) or specialised brainstem-optimised sequences to more precisely track brainstem function during chewing. Concurrent imaging modalities, such as combined MRI and electrophysiological recordings targeting trigeminal motor nuclei, could also shed light on the dynamic interplay between subcortical pattern generators and the robust cortical activation that accompanies human mastication.

In summary, fMRI evidence confirms that chewing gum engages extensive neural networks, prominently including motor, sensory, and certain cognitive–emotional circuits. These results bolster the notion that mastication is not purely reflexive but rather an integrated sensorimotor task capable of modulating arousal, stress responses, and possibly cognitive function. Ongoing research that refines imaging methodologies, explores different populations (e.g., older adults, clinical groups), and incorporates long-term interventions will help elucidate the broader impact of gum chewing on brain function and behaviour.

### 4.2. fNIRS Outcomes

**Figure 3:**
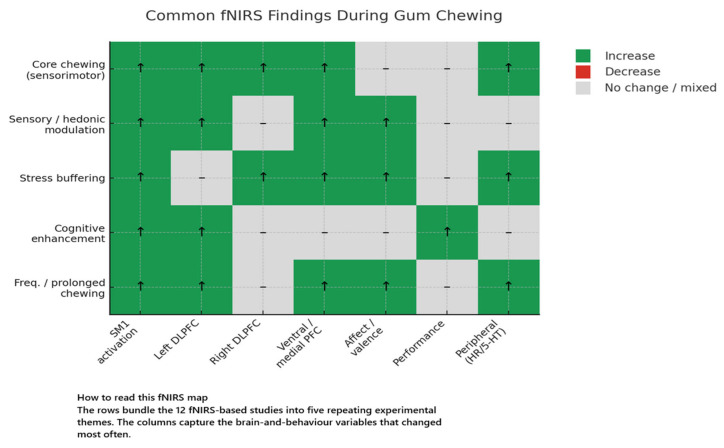
Common fNIRS findings during gum chewing.

The fNIRS studies [41,42,43,44,45,46,47,48,49,50,51,52] collectively demonstrate that gum chewing measurably affects cortical haemodynamics and related psychological or behavioural outcomes. A consistent finding is increased blood flow and oxygenation in the prefrontal cortex (PFC), a region vital for executive function, emotional regulation, and cognitive control. This PFC activation appears across varied experimental conditions, including different flavours, emotional contexts, and chewing frequencies. A description of the common findings based on fNIRS is presented in Figure 3.

#### 4.2.1. Flavour and Emotional Valence

Studies [41,42] highlight how gum’s gustatory and olfactory properties influence PFC activity and emotional responses. In study [41], participants chewed two gums with distinct valences: a palatable lemon-flavoured gum and an unpalatable salty licorice-flavoured gum. Subjective ratings confirmed the latter’s lower taste and odour appeal. Despite both conditions activating the bilateral primary sensorimotor cortex (consistent with the motor demands of chewing), the unpalatable gum selectively elicited heightened activation in the left frontopolar region and dorsolateral prefrontal cortex (DLPFC). This targeted increase in prefrontal haemodynamics suggests that negative or unpleasant flavours engage additional affective or cognitive resources—possibly reflecting an evaluative process in which the brain allocates more attention to aversive sensory input.

Study [42] expanded on this by comparing gums that varied in taste and odour separately: a control gum (no taste, no odour, C-gum), a sweet gum without odour (T-gum), and a sweet gum with a lemon odour (TO-gum). Participants rated the TO-gum as the most flavourful and the C-gum as the least. Concomitantly, transcranial doppler (TCD) and near-infrared spectroscopy (NIRS) showed that the TO-gum condition yielded significantly higher middle cerebral artery blood flow velocity (MCAV) and oxygenated haemoglobin (O2Hb) in the frontal cortex compared to T-gum and C-gum. Because the overall chewing effort (as measured by masseter EMG) did not differ among conditions, the additional increase in blood flow for the TO-gum was attributed to the amplified sensory load and associated hedonic or emotional processing—namely the synergy of taste plus odour. Together, these findings indicate that flavour intensity and emotional valence can upregulate PFC activation, with negative or complex multisensory flavours prompting greater neural resource allocation than simpler or more neutral ones.

#### 4.2.2. Motor Control vs. Cognitive/Emotional Processing

While gum chewing inherently engages sensorimotor pathways, including the bilateral primary sensorimotor cortex [41,42], multiple findings suggest that the additional PFC activation seen in these studies reflects more than just chewing effort. Crucially, when researchers measured masseter muscle activity to index chewing intensity, they often found no significant differences between conditions that still produced distinct PFC responses. For example, in study [41], participants chewed unpalatable versus palatable gum at the same rate and with similar muscle activation, yet the unpalatable condition elicited stronger haemodynamic signals in the left frontopolar area and dorsolateral prefrontal cortex (DLPFC). Similarly, in [42], participants’ masseter muscle activity remained comparable across three gum types, but the gum with both taste and odour (TO-gum) produced markedly higher cerebral blood flow than the other two. These discrepancies indicate that the sensory–emotional properties of gum, rather than differences in mastication force or rhythm, drive additional PFC engagement. Further underscoring this point, study [43] showed that imposing voluntary control over chewing side or rhythm did not differentially affect middle cerebral artery blood flow velocity, even though it altered muscle activation patterns. Whether participants chewed freely or restricted themselves to one side at a fixed rate, their cerebral circulation increased during mastication to a similar degree. The lack of divergence under varying motor constraints implies that purely motor-related factors (e.g., side of chewing, speed, muscle activation) are not the primary determinants of PFC activation. Instead, these findings collectively suggest that the cognitive and affective dimensions—such as flavour perception, novelty, or emotional valence—are key contributors to the heightened prefrontal responses observed across studies.

#### 4.2.3. Chewing Frequency and PFC Activation

Studies [48,49] systematically examined how altering chewing speed affects PFC activation. In study [48], participants performed three trials of gum chewing at 30, 70, and 110 chews per minute for 3 min each. NIRS measurements over the left PFC showed a significant rise in Oxy-Hb at all frequencies compared to baseline; however, chewing at 110 chews per minute led to a notably higher Oxy-Hb increase (*p* = 0.0003) than did chewing at 30 chews per minute. Although Oxy-Hb rose moderately at 70 chews per minute, it did not statistically differ from 30 chews per minute, highlighting a threshold-like effect in which faster chewing amplified cortical activation more robustly. Study [49] employed a similar three-tier design (30, 70, and 110 chews per minute for 5 min) but added a cognitive assessment—the Uchida–Kraepelin (U-K) Test—both before and after chewing. Consistent with [48], 110 chews per minute significant boosted PFC oxygenation compared to baseline pre-chewing levels (*p* < 0.05), while 30 and 70 chews per minute showed no significant changes. Despite this pronounced haemodynamic response at the highest chewing frequency, no corresponding improvement was observed in the U-K Test scores. This dissociation suggests that, while faster chewing intensifies PFC activation—likely through enhanced somatosensory feedback or heightened neuromuscular engagement—such physiological changes do not necessarily improve cognitive performance.

#### 4.2.4. Cognitive Performance Outcomes

Several studies investigated whether the observed increases in PFC activation during gum chewing translate into cognitive gains, yielding mixed results. In study [45], participants completed a Stroop test—an executive function task requiring quick, accurate responses to congruent or incongruent colour–word pairings—under chewing and non-chewing conditions. fNIRS revealed a significant rise in oxygenated haemoglobin (oxy-Hb) in the left DLPFC when participants chewed gum (*p* < 0.05). Correspondingly, reaction times were significantly shorter (*p* < 0.05) compared to the non-chewing condition, suggesting a facilitation of processing speed or attentional control. Notably, accuracy did not differ between conditions, implying that gum chewing selectively enhanced response speed without compromising task precision. By contrast, [52] used a serial recall task to test short-term memory performance, requiring participants to memorise and reproduce eight-digit sequences. Although chewing gum produced a pronounced, short-lived increase in oxy-Hb in the left DLPFC (*p* < 0.05), task accuracy and reaction times remained unchanged. Performance remained stable across all four conditions (before chewing, while chewing, immediately after chewing, and five minutes later), suggesting that heightened PFC activation did not confer a tangible memory advantage. Additionally, oxy-Hb levels returned to baseline within five minutes post-chewing, reinforcing that any neurophysiological benefit might be transient and task-dependent. A similar dissociation emerged in study [49], where chewing at 110 chews per minute robustly elevated PFC oxygenation but did not improve Uchida–Kraepelin test scores. Taken together, these findings imply that, while gum chewing can facilitate certain cognitive operations—particularly tasks demanding rapid response or executive control—its benefits may not extend to all domains of cognition, such as complex memory retrieval. The nature of the task (e.g., speeded executive function vs. recall-based tasks), the temporal window of heightened cortical activation, and individual differences likely all modulate whether an observable performance gain arises from gum-induced increases in PFC haemodynamics.

#### 4.2.5. Stress, Emotion, and Comfort

A subset of studies explored gum chewing as a modulator of stress and emotional reactivity, particularly during exposure to unpleasant or arousing stimuli. In studies [46,47], participants listened to negatively valenced sounds from the International Affective Digitized Sounds (IADS-2) database, which reliably elicit discomfort. These investigations used fNIRS to measure PFC activity alongside electroencephalography and autonomic indices (heart rate, subjective ratings). Despite differences in sampling and slight variations in methodology, both studies converged on the finding that gum chewing increased PFC activation beyond what was induced by the negative sounds alone. For instance, in [46], total haemoglobin (total-Hb) in the right PFC was elevated by the unpleasant auditory stimuli. Adding gum chewing further heightened that activation (*p* < 0.01), while simultaneously boosting alpha wave activity—a pattern often linked to a more relaxed or alert-yet-calm state. Participants also reported feeling less discomfort and exhibited a higher heart rate, indicating a mixed physiological response: although the heart rate rose (presumably reflecting arousal from mastication), subjective reports pointed to reduced stress when chewing gum. In [47], a similar protocol (negative sounds with or without gum) yielded parallel results. Chewing gum led to a greater increase in oxygenated haemoglobin (oxy-Hb) in the right medial PFC (*p* < 0.05) compared to no-chewing conditions. EEG alpha wave rates increased, and participants reported lower anxiety scores (as measured by the State-Trait Anxiety Inventory) and higher comfort ratings on a visual analogue scale (VAS). These findings suggest that, although gum chewing raises cortical activation and may even elevate heart rate, it exerts a psychologically soothing effect and bolsters top-down regulation of stress. Study [50] offered a complementary perspective where participants listened to pleasant sounds while either resting, chewing gum, walking, or walking plus chewing gum. Chewing—alone or with walking—significantly increased PFC activity relative to the control condition (*p* < 0.05) and yielded higher subjective pleasantness ratings. Notably, walking alone did not significantly increase PFC haemodynamics, underscoring that gum chewing itself may be the primary driver of these changes in both neural activation and mood enhancement. Taken together, these findings point to an intriguing dual effect: gum chewing intensifies PFC activity (reflected by increased haemoglobin signals) but also improves subjective comfort or reduces perceived stress under challenging conditions. This is likely due to a top-down regulatory mechanism in which heightened prefrontal engagement supports emotional regulation and coping responses, ultimately leading to a subjective sense of relaxation despite the concurrent physiological arousal of mastication.

#### 4.2.6. Pain Modulation and the Serotonergic System

Study [51] uniquely examined whether gum chewing exerts an analgesic effect by engaging the PFC and modulating serotonin (5-HT) levels. Ten healthy adults performed a prolonged (20 min) gum chewing task at a self-paced rhythm, during which researchers monitored both PFC haemodynamics and a nociceptive flexion reflex (NFR). The NFR, elicited by electrical stimulation of the sural nerve in the right ankle, provides an objective index of pain sensitivity through electromyographic (EMG) responses recorded in the biceps femoris. Concomitantly, serotonin concentrations in whole blood were measured via high-performance liquid chromatography (HPLC) at three time points: before, immediately after, and 30 min post-chewing. fNIRS data showed a selective rise in oxygenated haemoglobin (oxy-Hb) within the ventral PFC (*p* < 0.05), pointing to enhanced regional blood flow and neural engagement in this subregion—a site often implicated in affective and motivational aspects of pain processing. No corresponding increase was detected in the dorsal PFC, suggesting that chewing preferentially activates specific prefrontal circuits. Importantly, the lack of significant changes in deoxygenated haemoglobin (deoxy-Hb) ruled out generalised systemic circulation effects, reinforcing the conclusion that gum chewing directly heightens local cortical oxygenation in the ventral PFC. Behaviourally, NFR responses were significantly reduced both immediately and 30 min after the chewing task (*p* < 0.05), indicative of lowered pain sensitivity. Parallel to these observations, whole-blood serotonin levels rose by approximately 7.7% (*p* < 0.05) immediately post-chewing and remained elevated after 30 min, mirroring the sustained suppression of the NFR. Taken together, these findings suggest that gum chewing triggers a cascade involving ventral PFC activation and increased serotonergic transmission, ultimately dampening pain signalling through descending inhibitory pathways. This study thus highlights a direct link between repetitive mastication, prefrontal cortical engagement, and the serotonergic system, supporting a broader role for gum chewing in pain modulation and stress regulation. Several experimental studies have confirmed that chewing gum reduces pain levels [94,95,96,97,98,99].

#### 4.2.7. Influence of Dual Tasks and Movement

Study [50] examined the combined effect of gum chewing and moderate walking on PFC activation and subjective emotional responses. Eleven healthy adult males each performed four conditions in a block design: (1) rest while listening to pleasant sounds (control), (2) gum chewing (GCh) while listening to pleasant sounds, (3) walking at 4 km/h on a treadmill while listening to pleasant sounds, and (4) walking plus gum chewing (dual task) while listening to pleasant sounds. Each block consisted of a 30 s rest period, a 60 s task period, and a subsequent 30 s rest. Gum was tasteless, of moderate hardness, and participants chewed at a self-paced rhythm. A two-channel NIRS system measured PFC haemodynamics across conditions. Results showed that both gum chewing alone and the dual task of walking plus gum chewing elicited significantly increased PFC activation compared to the control condition (*p* < 0.05), whereas walking alone did not produce a significant change in PFC activity relative to rest. This suggests that moderate walking at 4 km/h may not be sufficiently demanding to recruit considerable prefrontal engagement but combining it with gum chewing imposes additional sensorimotor or attentional loads that elevate cortical activation. Subjective ratings of pleasantness collected via a visual analogue scale (VAS) were higher during gum chewing, whether performed on its own or alongside walking, indicating that the act of chewing—possibly by way of rhythmic oral somatosensory stimulation—boosts positive emotional states. The dual task maintained this effect without diminishment, suggesting additive or synergistic benefits for enjoyment. Taken together, these findings underscore how a seemingly simple behaviour like gum chewing can heighten PFC activation beyond what is observed during walking alone, and they illustrate that performing a dual task of light exercise plus chewing can enhance both cortical engagement and subjective pleasure without apparent interference effects.

#### 4.2.8. The Role of Gum Chewing on fNIRS Results in the Context of Brain Disorders

Dysfunction of the PFC occurs in many disorders, including major depressive disorder (MDD) [100,101]. FNIRS studies have identified altered PFC blood flow in MDD [102]. Chewing gum can reverse these changes in a lasting manner. Several studies have shown that chewing gum improves depression scores [8,103,104]. Furthermore, in dementia disorders, fNIRS results have also revealed abnormalities, including in the PFC [105]. The relationship between changes in adult brain blood flow and mastication as a preventive factor against cognitive decline was examined in a review of the literature [5]. This review showed that maintaining normal masticatory function by regularly chewing gum increases blood flow in the PFC, which may contribute to slowing cognitive decline. However, studies in this patient group are necessary to confirm this beneficial effect.

### 4.3. EEG Outcomes

**Figure 4:**
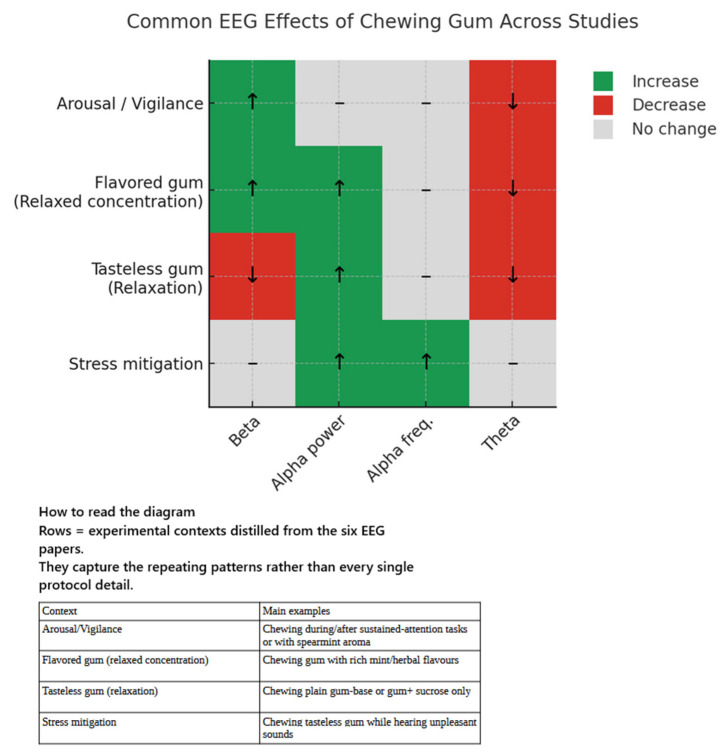
Common EEG effects of chewing gum across studies. Examples of studies: 1. Arousal/vigilance [53,54]; 2. Flavoured gum (relaxed concentration [56,57]; 3. Tasteless gum (relaxation) [56]; 4. Stress mitigation [46,47].

The seven EEG studies [46,47,53,54,55,56,57] indicate that chewing gum can transiently alter electroencephalographic activity and related measures of alertness in healthy adults. While the specific EEG changes vary depending on gum constituents, recording sites, and experimental protocols, several overarching themes emerge. A description of the common findings based on EEG is presented in Figure 4.

Study [53] reported that reaction times and vigilance were temporarily improved during and immediately after gum chewing, accompanied by increased beta power and higher heart rate, both common physiological indicators of heightened arousal [106,107,108,109]. In study [54], gum base alone and spearmint aroma conditions were linked to an increase in beta waves (at multiple scalp sites), again suggesting arousal or alertness. Similarly, study [56] found that flavoured gum (containing peppermint, lavender, lemon balm, chamomile, and lemon verbena oils) substantially increased alpha and beta power, indicating a more activated brain state than chewing an unflavoured gum base.

However, these effects were short-lived. Study [53] noted that performance and cortical activation gains faded by the second post-chewing session, aligning with a physiological rebound after mastication ceases. Study [55] likewise noted changes in specific frequency bands (e.g., increased alpha across multiple electrodes) right after chewing, but the effect was not evident at longer post-chewing intervals.

Several studies [54,56,57] highlight flavour and sugar as important modulators of EEG response. For instance, study [54] found that spearmint gum increased arousal (rise in beta, drop in theta), whereas chewing gum with sucrose could instead induce more relaxation (rise in theta, drop in beta). Study [56] similarly showed that standard (unflavoured) gum base led to an increase in alpha (often interpreted as lessened tension or mild relaxation [110]), whereas flavoured gum boosted both alpha and beta power, reflecting concurrent relaxation and mental alertness. Study [57] reported shifts in EEG source locations and Global Field Power (GFP) when participants chewed flavoured gum, along with increased subjective refreshment, suggesting that the taste and smell components help drive changes in brain arousal.

Across the studies, EEG changes were typically widespread (occipital, frontal, temporal, and parietal regions). This pattern indicates that gum chewing—especially when flavour or scent is involved—modulates multiple cortical areas, from sensory and emotional processing (temporal/frontal) to visual/spatial (occipital/parietal) regions. In study [53], electrodes on the left frontal (F7) and left temporal (T3) sites showed significant changes in beta power, which may reflect heightened cortical engagement needed for a vigilance task.

There are several possible mechanisms underlying chewing gum’s EEG effects.

Mastication activates the trigeminal nerve, which carries sensory information (e.g., pressure, texture) from the oral cavity to the brainstem (the trigeminal sensory nucleus) [111]. This sensory drive can modulate the ascending reticular activating system (ARAS), leading to increased cortical arousal measurable on EEG.

As seen in study [53], heart rate increases while chewing gum, suggesting mild sympathetic activation. Elevated autonomic arousal can feed back to cortical structures, amplifying beta-band activity. Enhanced blood flow or changes in respiration due to rhythmic mastication could also boost oxygenation in the cortex, thereby influencing EEG.

Flavourings (mint, citrus, etc.) stimulate olfactory and gustatory pathways, engaging limbic structures (e.g., orbitofrontal cortex, amygdala) that integrate reward, emotion, and arousal [112]. This contributes to the enhanced EEG beta or suppression of alpha that is typically correlated with alertness and engagement. Aromatic oils such as peppermint or lavender may also stimulate subcortical cholinergic and noradrenergic nuclei, reinforcing cortical activation.

Chewing gum can reduce mind wandering or direct attention to the chewing act itself, leading to improved task performance [113]. EEG signatures of focused attention often include beta oscillations in frontal areas and reduced theta [114]. Additionally, top-down control from the prefrontal cortex on sensory areas might be temporarily enhanced during gum chewing, yielding the boost in alpha observed in certain tasks, or altering alpha vs. beta coupling [115].

From a broader perspective, these findings reinforce that sensorimotor behaviours—even relatively simple ones like chewing—can have measurable effects on cortical dynamics. The short-lived nature of these enhancements points to practical applications (e.g., chewing gum during mentally fatiguing activities) but limited sustained benefit. Moreover, the flavour factor consistently shapes both EEG and subjective experience, suggesting that sensory cues and mastication interact to modulate arousal. Individual differences (e.g., caffeine intake, habitual gum chewing, or differences in flavour preferences) and task context (cognitive load, stress level, time of day) likely influence whether gum chewing leads to alertness or mild relaxation. Future research should use larger sample sizes, continuous EEG recordings during extended chewing periods, and high-density EEG arrays to clarify the spatial–temporal patterns of cortical changes. Ultimately, these studies highlight that chewing gum can produce short-lived increases in arousal and/or relaxation evident in the alpha and beta frequency bands, partly mediated by gustatory–olfactory stimulation, trigeminal input, and autonomic arousal. While the magnitude and duration of these effects vary, the common thread is that mastication—particularly with flavoured gum—can engage cortical circuits associated with vigilance, attention, and mood.

### 4.4. Chewing Gum and Alpha Oscillations: Mechanisms and Functional Significance

Chewing gum has been found to modulate brain activity, most consistently by increasing the power of EEG alpha oscillations (8–13 Hz). Multiple studies report elevated alpha power during and following mastication. For instance, chewing an unflavoured gum base increased alpha-wave power relative to baseline [56]. When flavour or aroma was introduced (providing mild sensory stimulation), chewing still enhanced alpha activity (often accompanied by a modest increase in beta power), producing what researchers describe as a “harmonious” state of simultaneous arousal and relaxation—termed “relaxed concentration” [56]. In other words, gum chewing appears to induce a calm yet alert brain state, marked by enhanced alpha rhythms. Understanding why chewing elevates alpha requires consideration of the underlying neurophysiological mechanisms, the psychological context of this effect, and theoretical models of alpha function.

Physiologically, alpha oscillations reflect dynamic interactions within thalamocortical networks and local cortical circuits that regulate neuronal excitability. The generation of the prominent ~10 Hz alpha rhythm is closely associated with synchronous activity in thalamocortical loops: neurons in the thalamic relay nuclei and the GABAergic thalamic reticular nucleus (TRN) can produce rhythmic bursting that entrains cortical alpha activity [116]. These oscillations effectively modulate cortical excitability in a periodic manner, functioning as a rhythmic gating mechanism for sensory input. In effect, the presence of a strong alpha rhythm indicates that cortical neurons are being periodically inhibited and excited in unison, controlling the flow of information via rhythmic changes in their responsiveness [117]. This supports the view that alpha activity provides “pulsed inhibition,” whereby each ~100 ms cycle imposes an inhibitory phase that reduces neural firing probability [118].

Chewing gum may engage these neural mechanisms through several pathways. The physical act of mastication sends patterned somatosensory signals (via trigeminal afferents to the brainstem) which can influence the ascending reticular activating system and thalamus, potentially biasing the brain toward an oscillatory state. Unlike highly novel or cognitively demanding stimuli, the rhythmic, repetitive nature of chewing produces a steady stream of predictable sensory input. This may encourage the thalamocortical circuit to settle into an idling oscillatory mode, rather than a desynchronised, high-arousal state. Supporting this, gum chewing does not typically heighten fast EEG activity (beta/gamma) associated with intensive processing; instead, it promotes alpha synchronisation. The result is a widespread increase in alpha power, indicating that cortical networks are in a more inhibited, less excitable state. Neurally, chewing-induced alpha may raise the threshold for neural firing—the brain becomes less reactive to irrelevant or trivial stimuli, as incoming input is gated by the prevailing oscillatory inhibition [117]. Such a state may be mediated by enhanced GABAergic tone in cortical interneuron networks entrained by the chewing rhythm, consistent with the “gating by inhibition” model (discussed further below), in which increased alpha reflects the active suppression of processing in certain neural pathways [118].

Another key neurophysiological aspect is large-scale network synchronisation. Alpha oscillations are not confined to one region; they can synchronise activity across distant cortical areas, binding together networks in a common rhythm [119]. During chewing, alpha enhancement may indicate that multiple brain regions (for instance, frontal, parietal, and sensorimotor areas) have become phase-locked in the alpha band. This synchronisation could facilitate coordinated “idling” or internal processing, as opposed to fragmented activity. Notably, studies of attention find that alpha-band phase synchrony can align activity in frontal and parietal cortices, effectively coordinating distributed neural processes [119]. In the context of gum chewing, increased alpha synchronisation might indicate that the brain enters a cohesive resting-state mode, operating smoothly and in unison, akin to an engine running in neutral. This idea corresponds to observations that alpha activity tends to increase in cortical areas that are not actively engaged by a task, while it decreases in regions that are processing task-relevant information [118]. Chewing gum, being a relatively automatic motor behaviour, does not heavily tax most cognitive systems; many cortical areas might thus enter an idling (yet synchronous) alpha state during chewing, especially in the absence of other demanding tasks.

#### 4.4.1. Psychological Interpretations

From a psychological perspective, the enhancement of alpha power during chewing gum is often interpreted as a marker of relaxation and stress reduction. Alpha waves are typically associated with tranquil wakefulness, becoming more prominent when an individual is calm and not actively engaged in demanding cognitive activity [120]. The observation that chewing gum elevates alpha suggests it can induce a more tranquil mental state. Indeed, chewing research has documented stress-buffering effects: it attenuates physiological stress responses and improves mood. For example, masticating under stress blunts the rise of stress hormones (cortisol and adrenaline) and reduces anxiety-related responses in both animals and humans [16]. Consistent with this, human studies report that gum chewers often experience lower perceived stress and improved mood during cognitive tasks [121]. The EEG signature of increased alpha aligns with these observations, since higher alpha is correlated with lower anxiety and greater feelings of calmness or contentment [122]. This alpha-dominated state likely underpins the stress-relieving and mood-enhancing effects attributed to chewing gum.

In addition to relaxation, alpha enhancement during chewing has implications for attention and alertness. Notably, elevated alpha in this context does not signify drowsiness (which would be characterised by slower theta waves); rather, a state of relaxed concentration. Gum chewing can produce a mental condition in which one is alert but not agitated. Behavioural studies have found that chewing gum can improve aspects of sustained attention: for instance, reaction times are often faster and vigilance (hit rates) increases during prolonged tasks when gum is chewed [53]. At the same time, chewing tends to prevent over-arousal or mental strain. It causes only a mild physiological activation (a slight increase in heart rate) and only transiently boosts high-frequency EEG activity (e.g., a modest beta increase at frontal sites immediately post-chewing) [53]. This combination of effects—simultaneous calmness and alertness—can be described as a form of attentive disengagement. The brain is not hyper-focused on any single external stimulus (hence the elevated alpha, reflecting sensory disengagement [118]), yet the person remains awake and responsive. By filtering out distractions and stress (via alpha-mediated inhibition) whilst providing a gentle arousal boost (via chewing’s stimulatory effects on the autonomic nervous system), gum chewing may optimise internal conditions for sustained attention. The alpha enhancement suggests that the chewer’s mind is buffered from irrelevant inputs or intrusive thoughts, reducing cognitive overload. Meanwhile, the subtle arousal ensures that this relaxed state does not drift into sleepiness. This psychophysiological balance aligns with the idea of “relaxed concentration”—a mental state that chewing gum seems uniquely capable of producing.

#### 4.4.2. Theoretical Models Integrating Alpha’s Role

Several theoretical frameworks help explain the alpha enhancement observed from chewing gum by situating it within the broader functional role of alpha oscillations.

1. “Gating by Inhibition” Framework: Alpha oscillations are thought to actively gate information flow in the brain by inhibiting neural processing in specific pathways or regions [118]. Jensen and Mazaheri formalised this idea, proposing that alpha activity reflects functional inhibition of cortical areas not currently required for the task at hand [118]. From this perspective, increased alpha power is not a passive byproduct of idling but an active process for filtering out distractions or irrelevant inputs. Chewing gum’s induction of alpha can thus be interpreted as a gating mechanism: rhythmic alpha oscillations suppress extraneous sensory input and stress-related signals, effectively “closing the gate” on noise and allowing the brain to operate more efficiently on pertinent matters (or to rest without intrusion). This inhibition is likely mediated by GABAergic interneurons, which generate rhythmic inhibitory postsynaptic potentials that periodically hyperpolarise cortical neurons. The gating by inhibition model aligns well with the gum-chewing findings—as one chews, alpha elevations in networks unrelated to the mastication task indicate those regions are being functionally inhibited, which can conserve resources and promote a stable, calm focus. Notably, when gum is chewed during a cognitive activity, alpha may increase in areas not directly engaged by the task, potentially preventing distraction by suppressing activity in task-irrelevant regions.
2. Alpha as an “Idling” Rhythm: Historically, alpha waves have been considered the brain’s default idling oscillation during wakefulness in the absence of demanding cognitive processing. Berger’s classic observation showed that alpha dominates the EEG during quiet rest (e.g., eyes-closed relaxation) and diminishes with mental engagement. In the context of chewing gum, part of the alpha increase may reflect an idling of certain cognitive systems. Chewing is a habitual, automatic motor behaviour that requires minimal conscious effort, allowing large portions of the cortex to enter an idle mode. For example, if no complex task is being performed whilst chewing, the visual cortex may exhibit strong alpha activity (akin to an eyes-closed state) due to a lack of critical visual input. Gum chewing facilitates reversion to a default, idling oscillatory state. However, modern interpretations emphasise that “idling” is not passive or inefficient; rather, it reflects the brain actively disengaging certain networks. Alpha, as previously noted, increases in regions not engaged by the current behaviour [118]—the neural correlate of an idling state. Thus, what was historically understood as an idle rhythm can be reinterpreted as the brain actively inhibiting unnecessary processing. Chewing-induced alpha may therefore represent a functional idling: the brain is awake and primed but in a low-engagement, energy-conserving mode. This state is adaptive for relaxation and recovery, and may also set a favourable baseline for switching to focused processing, as the brain in an alpha-rich idle state can rapidly desynchronise and reallocate resources upon salient stimulus or task demands.
3. Inhibition-Timing Hypothesis: Building upon the above concepts, Klimesch and colleagues proposed an “inhibition-timing” model of alpha oscillations [123]. They posit that alpha rhythms reflect periodic windows of cortical inhibition, precisely timed to regulate neuronal firing. The oscillation’s phase and amplitude modulations serve as a timing mechanism for neural excitability. Notably, alpha power often increases (event-related synchronisation, ERS) in situations requiring top-down control, such as when a person must withhold a response or endure a delay—conditions in which the brain imposes inhibition to prevent premature or irrelevant activity. Thus, alpha ERS is interpreted as an active top-down inhibitory control process. The timing aspect of the hypothesis emphasises that the rhythmic nature of alpha (e.g., ~10 cycles per second) creates alternating periods of inhibition and disinhibition in neuronal populations, effectively dividing time into discrete windows where processing is suppressed versus allowed. Empirical support for this comes from findings that alpha phase coherence between brain regions increases during tasks requiring coordination, with phase lags consistent with neural transmission delays [123]. In practical terms, the inhibition-timing model means that alpha oscillations can temporally organise neural firing. Applied to chewing gum, it can be hypothesised that the act of chewing engages such inhibitory timing mechanisms to optimise neural processing. The brain might use alpha oscillations to periodically inhibit irrelevant sensory inputs or motor impulses in synchrony with the chewing cycle. For instance, if an individual is chewing while also performing a task, alpha could help “schedule” processing by providing brief inhibitory epochs that protect the ongoing task from interference at regular intervals. Even in the absence of an external task, the very act of rhythmic chewing could entrain alpha-timed inhibition across sensorimotor and associational areas, which would manifest as the observed alpha power increase. The inhibition-timing framework therefore enriches the explanation by suggesting that chewing-induced alpha is not just a byproduct of relaxation, but also an indicator that the brain is actively timing its inhibitory control of neural processes. This could be one reason why the alpha state during chewing still permits responsiveness and does not equate to sleep—the timing of inhibition is organised in an optimal way to maintain readiness.
4. Predictive Coding Model: Recent theoretical work has linked alpha oscillations to predictive coding, a mechanism by which the brain continually predicts incoming sensory inputs and minimises surprise. In predictive coding models, higher-level neuronal regions send predictions (or suppressing signals) to lower-level sensory areas to eliminate expected input, allowing the brain to focus on unexpected or novel information. Alpha-band activity has been proposed as a carrier of these top-down predictions, acting to silence or filter out expected stimuli [124]. This framework elegantly accounts for the increase in alpha power observed during gum chewing. Chewing is a self-generated, rhythmic act that produces highly predictable sensory consequences (e.g., the feel of jaw movement, the taste and texture of gum, etc.). The brain quickly learns this pattern and can anticipate the sensory feedback from each chew. According to predictive coding theory, the brain sends inhibitory predictions to the somatosensory and other relevant cortices to dampen responses to expected chewing-related input. Alpha oscillations are a plausible mechanism for implementing this inhibitory prediction—by oscillating in phase with the predicted sensory events, they could suppress neural responses at the optimal moment. The result would be an overall increase in alpha power during chewing, reflecting the fact that much of the incoming information (from the chewing motions) is predicted and gated out. This minimises “surprise” or prediction error, contributing to the subjective feeling that the act becomes automatic or mindless. In short, the predictive coding model suggests that chewing-induced alpha is a sign that the brain’s internal models are effectively accounting for the sensory consequences of chewing, thereby silencing redundant inputs. This perspective complements the gating-by-inhibition framework: it suggests that alpha-mediated predictive suppression is the specific means in which the brain closes the gate on expected information. By doing so, the brain remains in a steady, relaxed state (since nothing unexpected is happening during rhythmic chewing), yet it is still prepared to detect any deviance. If, for instance, an unexpected stimulus occurs, alpha would momentarily diminish to allow for error detection. In this manner, alpha oscillations during chewing exemplify the brain’s predictive regulation of its sensory environment.

In summary, the increase in alpha power associated with chewing gum can be interpreted as the combined outcome of multiple neurophysiological and functional mechanisms. Chewing activates thalamocortical circuitry that promote rhythmic synchronisation and neural inhibition, resulting in elevated alpha-band activity and reduced cortical excitability. Psychologically, this corresponds to a state of calm alertness—a relaxed yet attentive mindset that buffers stress without induing hypervigilance—“relaxed concentration”. Theoretical models converge in explaining this phenomenon: alpha oscillations during chewing gum serve to inhibit irrelevant neural processes (gating by inhibition), reflect an organised idling or standby mode of the cortex (idling hypothesis), impose a temporal structure on neural inhibition (inhibition-timing hypothesis), and implement top-down predictions that attenuate expected inputs (predictive coding). Through these intertwined mechanisms, rhythmic chewing—a seemingly simple behaviour—can entrain the brain into a unique oscillatory state associated with relaxation, efficient information processing, and enhanced resilience to stress. In this alpha-dominant state, the brain is = “idling in gear”: energetically relaxed and synchronised, yet poised to respond when necessary, owing to the inhibitory timing and predictive regulation mediated by alpha rhythms. This integrative account not only explains how chewing gum can increase alpha power, but also highlights the broader significance of this effect for the organism’s cognitive and emotional functioning.

### 4.5. The Effects of Flavours in Chewing Gum—Additional Changes in Brain Activity and Possible Therapeutic Potential

Within this review, studies employing flavoured gum consistently reported significant alterations in brain electrical activity compared to unflavoured gum. For instance, in study [56], chewing flavoured gum (a blend of peppermint, lavender, lemon balm, chamomile and lemon verbena) elicited distinct effects not observed with unflavoured gum. Alpha activity significantly increased across a broader range of sites, including parietal (P3, P4, Pz), temporal (T4), frontal (F4), and prefrontal locations (Fp2), indicative of enhanced cortical synchrony. Beta activity also increased significantly at Fp1, O2, and T3—contrasting with the decrease observed in the non-flavoured condition. In addition, theta activity significantly decreased across nearly all recorded sites (O1, O2, P3, P4, T3, T4, F3, F4). Inhalation of the same aromatic oil (applied to gauze) significantly increased alpha power at P3, F3, and Fz, and beta power at P3 and Fz. No significant changes were observed in the theta or delta bands. In study [54], inhaling spearmint aroma decreased alpha activity at O2, F3, P4, and Fz, while increasing beta activity at the same sites, indicating a stimulating effect. In contrast, chewing gum base containing sucrose increased theta activity at Fp2 and Fz and decreased beta activity at Fp1, Fp2, F4, P3, P4, Fz, and Pz, suggesting a relaxing effect. Sucrose ingestion alone did not significantly alter EEG activity. In study [57], two flavoured gums were compared: one was based on the standard gum base and included various sugars (72%), herbal essence oils (primarily valerian, liquorice, and 1% lavender), and perfumes (lemon, peppermint, and 2% lavender); and a “special gum” containing theanine. Post-hoc analysis of EEG source models revealed differential shifts. The alpha-2 source model exhibited an anterior shift after chewing regular gum but a posterior shift after gum base, with corresponding rightward (regular gum) and leftward (gum base) shifts. The beta-2 source model shifted inferiorly after regular gum and superiorly after gum base, with a tendency towards anterior shift after regular gum and a posterior shift after gum base. Regarding global field power, in all three bands (delta-theta, alpha-2, and beta-1), there was an increase for regular gum and a decrease for gum base. Study [42] used NIRS and showed that chewing sweet-tasting gum combined with lemon odour (TO-gum) produced the most pronounced bilateral increases in DO_2_Hb concentrations compared to taste-only gum (T-gum) and control gum (C-gum). This increase began approximately two minutes after chewing onset, peaked at approximately three minutes, and notably persisted for about two minutes after chewing had ceased, especially in the left frontal lobe. In contrast, chewing the T-gum and C-gum resulted in smaller, statistically non-significant increases in DO_2_Hb during the chewing phase, although minor post-chewing elevations were observed. Investigating olfactory-induced brain responses [36] exposed participants to three gum odours: L-menthol (a minty, trigeminal), L-carvone (minty, non-trigeminal), and strawberry (non-trigeminal, non-minty). When analysing the brain response to the trigeminal component (L-menthol > L-carvone), the high-frequency group demonstrated stronger activation in the midcingulate cortex, postcentral gyrus, precentral gyrus, supplementary motor area (SMA), inferior parietal lobule, insula, and anterior cingulate cortex. Conversely, the low-frequency group exhibited greater activation primarily in the superior temporal lobe and insula. Additionally, in the low-frequency group, enhanced brain responses to L-carvone > L-menthol were observed in olfactory-related regions, including the orbitofrontal cortex, hippocampus, and anterior cingulate cortex. Between-group comparisons confirmed that the high-frequency group exhibited stronger activation than the low-frequency group in response to trigeminal stimulation (L-menthol > L-carvone), particularly in the right SMA, bilateral paracentral lobule, right precentral gyrus, and precuneus. No superior activation was found for the reverse contrast in the low-frequency group. For the minty sensation (L-carvone vs. strawberry contrast), there was no significant group × odour interaction in brain activation.

Aromas exert complex and varied effects on brain activity. Sayorwan et al. reported that lavender oil increased theta (4–8 Hz) and alpha (8–13 Hz) power [125]. However, conflicting findings exist, and another study found a significant decrease in alpha-1 (8–10 Hz) EEG power in the parietal and posterior temporal regions soon after the onset of lavender oil inhalation. Significant alpha-1 changes were also observed after inhalation of eugenol or chamomile [126]. Diego et al. documented that lavender aromatherapy increased beta power, while rosemary reduced both frontal alpha and beta power [127]. In the study of Sayowan et al., after the inhalation of jasmine oil, the beta wave power (13–30 Hz) was increased in the anterior centre as well as the left posterior region [128]. Lin et al. [129] observed that exposure to peppermint aroma increased alpha activity in the prefrontal area. Beyond EEG, NIRS measurements by Yakugaku et al. during N-back task performance indicated that lavender aromatherapy (fragrance of lavender) significantly increased regional blood flow in the inferior frontal cortex, compared to a no aroma condition [130].

The results from studies using aroma exposure show overlap with those found in gum chewing studies. Peppermint aroma in chewing gum has been shown to increase alpha power, but inhaling the same aroma alone has the opposite effect [56], suggesting that the method of exposure matters.

Taken together, these findings suggest that future research should examine chewing gums with different flavours. Such studies could confirm whether or not flavours (and certain types) elicit more pronounced effects on brain activity compared to unflavoured gum. It would also be worth verifying the effect of gums with specific essential oils, particularly lavender, jasmine, and peppermint. This line of inquiry is particularly pertinent given the established efficacy of aromatherapy as a valuable and effective intervention in alleviating symptoms of depression [131,132], anxiety [133], or improving cognitive functions [134]. Beyond immediate brain responses, future studies employing flavoured chewing gums, in addition to further examining the immediate and long-term effects, should also explore their potential for improving functional and behavioural outcomes, as studies on aromatherapy have shown. Positive results in this domain may encourage manufacturers to develop and market these types of gums, which will have a proven beneficial effect on human well-being and functioning.

## 5. The Need for Further Investigation into the Therapeutic Potential of Gum Chewing

The studies reviewed here agree that mastication changes brain function, from robust BOLD increases in sensorimotor and limbic circuits to frequency-specific modulations of EEG power and connectivity. By contrast, relatively few experiments have paired those neural read-outs with rigorous behavioural endpoints; the available behavioural literature suggests only modest improvements in stress ratings, alertness, and selected memory or attention measures. Because almost all of those behavioural trials were run without any concurrent imaging, the field still lacks a mechanistic bridge between “how gum chewing feels” and “what gum chewing does in the brain.” Future protocols must integrate both levels of measurement to reveal whether the neural signatures reported so far actually mediate the observed psychological benefits.

Gum chewing is also attractive because it is a light, hands-free activity that can be performed during other tasks. In clinical neuroscience, the best-established “hands-free” modulators are non-invasive brain-stimulation (NIBS) techniques such as transcranial direct-current stimulation (tDCS) and repetitive transcranial magnetic stimulation (rTMS); both are routinely delivered while participants sit, read, or perform cognitive exercises [135,136,137]. Because tDCS/rTMS target cortical excitability and functional networks [138,139,140], and because participants could in principle chew gum during the stimulation period, it is logical to test a combined gum-chewing + NIBS intervention. Evidence from other paired paradigms—e.g., cognitive training executed concurrently with anodal tDCS—shows additive or even supra-additive gains on working-memory accuracy [141], supporting the plausibility of a synergistic mastication-plus-neuromodulation effect for cognition or mood.

Whether chewing alone induces durable neuroplastic change remains uncertain: almost all imaging studies have probed only the minutes immediately before, during, or after mastication. Nevertheless, longitudinal behavioural and physiological findings (e.g., improved executive scores in older adults after weeks of masticatory exercise [142], or sustained rises in brain antioxidants after repeated hard-chewing bouts [2]) hint that plastic adaptation is possible. If so, augmenting those peripheral-to-central signals with a plasticity-promoting neuromodulation protocol could magnify and stabilise the benefits.

Finally, gum chewing should be benchmarked against other accessible, non-pharmacological therapies that target similar endpoints. Cognitive training paired with neurofeedback enhances memory and attention [143], mindfulness-based cognitive therapy and yoga yield medium-to-large reductions in anxiety and depressive symptoms [144,145]. Direct head-to-head trials (e.g., gum vs. mindfulness for acute stress, or gum + tDCS vs. neurofeedback for working-memory load) will clarify whether mastication is merely convenient or genuinely competitive with these established interventions.

In short, chewing gum is a low-cost, portable behaviour that modulates neural activity; pairing it with simultaneous behavioural read-outs, modern neuro-imaging and, potentially, synergistic neuromodulation will be essential to establish its therapeutic value.

## 6. Limitations and Future Directions

Despite a growing body of evidence indicating that chewing gum modulates both regional brain activation (via fMRI/fNIRS) and cortical oscillations (via EEG), several methodological and interpretational caveats persist across these studies. Below, the key limitations are summarised and proposals for directions of future work are proposed.

### 6.1. fMRI Studies

#### 6.1.1. Sampling and Generalisability

Across the fifteen fMRI papers reviewed, participant pools were uniformly modest—median size sixteen, with only two studies exceeding thirty volunteers—which means statistical power is limited, effect sizes are likely inflated, and meaningful subgroup contrasts (for instance, sex-by-age interactions) are impossible. Age distribution is heavily skewed toward young adults; fourteen of the studies sampled almost exclusively from the 19-to-35-year range, while just one project included middle-aged and older-old cohorts and, tellingly, revealed pronounced age-related shifts in activation that escape the youthful profiles dominating the field. Handedness restrictions further narrow the lens: virtually every experiment enrolled right-handed individuals only; the single exception that juxtaposed five right- with five left-handers left ambidextrous participants unrepresented even as several papers linked chewing-side preference, lateralised BOLD patterns, and perfusion changes to dominant-hemisphere circuitry. Although most groups reported roughly balanced male–female ratios, the absolute numbers were too small to interrogate sex effects, and no protocol considered hormonal status, menstrual cycle, or menopausal stage, despite documented sex differences in orofacial muscle strength [146,147] and pain sensitivity [148,149,150]. Attrition caused by head-motion cut-offs compounds the sampling problem; exclusion rates reached nearly half the initial cohort in one study, systematically purging precisely those individuals—people with dental prostheses, chronic orofacial pain, bruxism, neurological impairment—whose chewing behaviour is clinically informative yet harder to stabilise in the scanner. Equally important, cultural, dietary, and occupational diversity is almost absent: convenience samples of students or hospital staff from East Asia, Europe, or North America dominate, with no reporting of ethnicity, habitual diet hardness, or chewing-intensive occupations, all factors that shape masticatory musculature and could modulate central representations. Finally, developmental and patient populations—children, adolescents, individuals with temporomandibular-joint disorders, post-stroke dysphagia, neurodegenerative disease, or post-orthodontic rehabilitation—remain entirely unstudied, even though understanding mastication’s neural basis is arguably most relevant for these groups. The next generation of chewing-fMRI should be carried out by a coordinated, pre-registered consortium of at least five MRI centres distributed across three continents—say, East Asia, Europe, and the Americas—to dilute local diet and scanner idiosyncrasies while capturing cultural, genetic, and gastronomic diversity. Each site would run an identical protocol that includes the same bite-force mouthpiece, prospective head-motion tracking, and stimulus sequence, ensuring true data pooling rather than a meta-analysis of heterogeneous studies.

In the future studies, recruitment must be stratified into five age bands—children (6–12 years), adolescents (13–17 years), young adults (18–35 years), middle-aged adults (36–60 years), and older adults (61–80 years)—with roughly one hundred participants in each bracket so that developmental trajectories and ageing-related compensation can be modelled explicitly. Every age stratum should be balanced for sex (50% female) and accompanied by detailed hormonal metadata such as menstrual phase, contraceptive use, or menopausal status, allowing the field to test long-suspected oestrogenic modulation of orofacial motor control. Hand dominance must also be diversified: at least one-quarter of all participants should be left-handed or ambidextrous, providing sufficient leverage to examine hemispheric lateralisation without the dominant-hemisphere bias that currently permeates the literature.

Parallel to the healthy sample, the project should oversample key clinical groups at translational stake—temporomandibular-disorder pain, post-stroke dysphagia, early Parkinson’s disease, and mild cognitive impairment—by recruiting a minimum of thirty patients per diagnosis and matching them to controls on age, sex, and handedness. This arrangement would for the first time allow direct case–control tests of whether mastication networks are merely altered or instead compensatorily up-regulated in pathology. All participants, clinical and healthy, must complete a standardised questionnaire quantifying habitual diet texture (soft, mixed, hard), weekly gum use, and any chewing-intensive occupation such as carpentry or tailoring; these variables can then be incorporated as covariates to disentangle state-related activation from lifetime masticatory load.

Finally, the statistical architecture should rest on a hierarchical Bayesian model that treats scanning site as a random effect and estimates fixed effects and interactions for Age × Sex × Handedness × Clinical Status. Pre-registered quality-control thresholds—≤1 mm framewise displacement, verified bite-force sensor readings, and intact stimulus logs—must be enforced uniformly, and both raw and pre-processed data should be deposited in an open repository. Implemented in this fashion, chewing-fMRI would progress from boutique physiology toward a population-relevant science capable of revealing developmental, hormonal, and pathological deviations in the human mastication network.

#### 6.1.2. Task Design and Experimental Control

The way “chewing” is operationalised varies so widely across the fifteen fMRI protocols that cross-study comparison becomes precarious. Motor prescription is the first source of variance. Most groups instructed a fixed rhythm of 1 Hz (studies 26, 27, 28, 29, 34, 38), but two allowed a broader 1–2 Hz window (study 30) and three permitted self-paced or “spontaneous” chewing (studies 31, 32, 37). Apart from a single EMG-monitored one-hour mastication exercise (study 40), compliance was assumed rather than recorded; no data logs confirm whether subjects actually hit the target frequency or force.

Bolus material and texture differ sharply. Five experiments used a moderately hard laboratory gum (5.6 × 10^4^ poise), one contrasted that with a hard version (2.3 × 10^5^ poise), another compared soft chewing gum with hard “wine gum”, one replaced gum by rubber-dam strips, and two allowed volunteers to bring their favourite commercial gum. Yet no study quantified bite force or bolus deformation in situ; hardness is therefore a nominal rather than calibrated variable, and flavour, sweetness, and trigeminal intensity are uncontrolled confounds except where explicitly manipulated (study 36).

Laterality and jaw-use instructions are equally inconsistent. Two studies forced unilateral right-side chewing (studies 28, 31), two alternated right and left (study 30) or determined side by chewing-side preference (study 40), whereas the remainder allowed bilateral mastication. Because hemifacial motor drive, jaw kinematics, and cortical lateralisation differ between ipsilateral and contralateral chewing, this heterogeneity clouds interpretation of hemispheric effects.

Baseline conditions diverge conceptually. “Rest” sometimes meant holding gum passively in the cheek (studies 28, 29); elsewhere, subjects adopted centric occlusion with no gum (study 30), or engaged in sham-chewing jaw movements without bolus (study 32). These baselines have very different proprioceptive and somatosensory loads, yet most papers treat them as interchangeable.

Block-design parameters range from 21 s epochs repeated three times (studies 28, 29) to 32 s epochs repeated eight times (studies 26, 38) and 20 s epochs repeated eighteen times (study 37). Only one project subdivided blocks into 5 s bins to look at initiation versus maintenance phases (study 31); none implemented truly event-related or jittered designs that would separate preparatory, rhythmic, and termination components or allow de-convolution of chewing from inevitable swallowing and salivation events.

Task order and randomisation are rarely handled rigorously. About half the studies always started with a rest block and alternated in a fixed sequence, risking predictability and physiological drift. When multiple chewing conditions were compared (e.g., hard vs. soft gum), the order was fixed in five of seven cases to minimise scanner-bed manipulation, at the cost of uncontrolled order effects.

Finally, concurrent cognitive loading differs dramatically. Two papers combined chewing with finger movements (study 28) or an Attentional Network Test (study 35), thereby engaging additional motor-cognitive circuitry, whereas the others used chewing as the sole task. Yet only one study (study 32) included a sham-chewing control to parse the cognitive contribution of “chewing imagery” from actual mastication.

Chewing research will only become cumulative when every laboratory measures the same physical act with the same hardware and stimulus taxonomy. To that end, each site in the consortium should adopt an MRI-compatible “chew kit” built around three core sensors. First, a thin piezo-resistive bite-force transducer—essentially a flexible polymer strip with a full Wheatstone bridge—must be embedded inside a tasteless, odourless gum puck. The strip should span both molar rows so that vertical force is sampled at 1 kHz with 0.5 N resolution, digitised via fibre-optic cabling to avoid radio-frequency loops. Second, jaw kinematics should be captured by a pair of retro-reflective markers glued to the lower incisors and tracked by an infra-red camera integrated into the head coil; the camera’s frame rate (120 Hz) will resolve the 1–2 Hz chew rhythm while remaining invisible to the EPI sequence. Third, a short bipolar surface-EMG lead placed over the right masseter—shielded, carbon-wire, and routed through the penetration panel—will furnish a myoelectric envelope that can later be convolved with a finite-impulse basis set when modelling trial-wise neural activity. A simple microcontroller synchronises all three channels and inserts TTL pulses into the scanner’s trigger line, guaranteeing sub-millisecond alignment between physiology and fMRI volumes.

With hardware unified, the behavioural protocol can at last be standardised. Each participant should complete a single 22 min run consisting of twelve 16 s chewing epochs separated by 16 s baselines, but the crucial twist is that gum hardness, laterality, and pace are parametrically stepped within-subject rather than across studies. The first four epochs use a soft bolus (35 kPa elastic modulus) at 1 Hz bilateral chewing; the next four switch to a hard bolus (120 kPa) still at 1 Hz; the final four keep the hard bolus but instruct a 2 Hz pace cued by an auditory metronome. Left-versus-right side chewing is counter-balanced across participants by instructing unilateral placement and verifying compliance in the kinematic trace. Because bite force and EMG are logged continuously, post hoc modelling can treat actual force, not nominal hardness, as a parametric regressor, thus disentangling mechanical load from flavour or cognitive set.

Baseline definition must also be uniform: subjects hold the current gum motionless against the left buccal mucosa with teeth parted 2 mm, an instruction that minimises proprioceptive noise yet preserves intra-oral flavour stimulation, preventing confounds tied to sweeteners leaching into saliva. To control for swallowing, an optical microphone taped under the larynx flags each deglutition; those fMRI time points are censored or entered as nuisance events.

#### 6.1.3. Motion and Spatial Resolution Constraints

Chewing is an unusually motion-laden fMRI paradigm: every 1 Hz jaw cycle generates millimetre-scale mandible excursions, propagates vibration through the skull base, and modulates magnetic susceptibility around the oral cavity. Yet the majority of the fifteen studies relied on conventional rigid-body realignment alone, with no prospective correction or model-based scrub of the rhythmic artefact itself. Head-movement ceilings spanned two orders of magnitude—from the stringent 0.75 mm translation cap in the original Onozuka protocol (study 26) to a 3 mm/1.8 mm *z*-axis limit in the cerebellar CPG search (study 37).

Spatial resolution is limited on two fronts: field strength and voxel size. Nine of the fifteen experiments were still performed at 1.5 T, with in-plane pixels of 3.75–4 mm and slice thickness 4–5 mm (effective volume ≈ 60–75 μL). Even the 3 T studies typically used 3–3.8 mm isotropic voxels to maintain reasonable SNR under the severe susceptibility gradients created by the maxillary sinuses; no protocol employed multi-echo EPI, parallel imaging acceleration, z-shimmed slices, or high-order shimming to salvage signal in the orbitofrontal cortex or brainstem. As a result, subcortical and infratentorial structures—precisely where the putative masticatory central pattern generator, trigeminal nuclei, and flavour circuits reside—are inconsistently detected or lost altogether. For instance, study 37 purposefully targeted brainstem CPG activity but ultimately reported no significant brainstem clusters and attributed the null finding to susceptibility dropout; study 40 circumvented the problem by abandoning BOLD EPI in favour of arterial-spin-labelling perfusion, but at a coarse 3.2 × 3.2 × 5 mm resolution.

Coverage concessions further compound the issue. Several early protocols (e.g., study 29) acquired only 20 axial slices centred on the temporal lobes, omitting cerebellum entirely. Where full-brain coverage was achieved (e.g., study 31, 53 slices), the long TRs (2.5–4 s) and thick slices trade temporal and spatial fidelity for coverage, blurring rapid onset/offset responses and hindering laminar or cerebellar–cortical connectivity analyses.

Finally, physiological noise modelling is virtually absent. None of the papers recorded cardiac or respiratory traces for RETROICOR or RVT correction, despite mastication’s tight coupling to breathing and the susceptibility of lower brain areas to pulsatile artefacts. Only one study (28) recorded surface EMG, but the signals were used for compliance assurance, not for multi-echo denoising or informed regressors.

In aggregate, the existing chewing-fMRI literature operates at the edge of what legacy EPI can tolerate, sacrificing spatial detail and statistical power to residual motion and susceptibility artefacts exactly where the neural circuitry of greatest interest is located. Methodological upgrades—prospective motion tracking, slice-accelerated multi-echo 3 T or 7 T imaging, advanced shimming and physiological noise regression—are essential prerequisites for confident mapping of the deep and infratentorial nodes of the mastication network.

#### 6.1.4. Temporal Resolution

Chewing unfolds on the scale of single jaw cycles (~1 Hz) and sub-second sensorimotor feedback loops, yet the temporal sampling schemes adopted in the fifteen studies are coarse and inflexible. Repetition times (TRs) range from 2.0 s (study 29) to 4.0 s (studies 26, 34, 37, 38), with a modal value of 3 s. At these TRs, the Nyquist limit is 0.25–0.5 Hz, so the primary 1 Hz motor rhythm is aliased into low-frequency noise; any attempt to model cycle-by-cycle dynamics is mathematically impossible.

The block architecture is equally sluggish: nine papers used 24 to 32 s blocks repeated three to eight times; two opted for 20 s blocks repeated 18 times (study 37) or 25 s blocks repeated ten times (study 31). With such long, predictable epochs, the canonical haemodynamic response inevitably plateaus, masking transient neural events linked to initiation (jaw opening, bolus placement), maintenance (rhythmic occlusion), and termination/swallowing. Only one investigation (study 31) partitioned its 25 s chewing block into non-overlapping 5 s windows to glimpse temporal evolution, but the underlying acquisition still had a 2.5 s TR—yielding barely two samples per window and poor de-convolution fidelity.

No study deployed an event-related or jittered rapid-sampling design that would allow separation of successive elements such as metronome cue, first bite, adaptive rate changes, inadvertent swallows, or brief pauses. Likewise, chewing-locked physiological regressors (e.g., convolving EMG-derived bite events with a finite-impulse response basis set) are absent; instead, task modelling relies on simple boxcar functions that assume a steady state within each long block. This simplification ignores known variability in bite force and inter-bite interval within a single chewing bout, effectively treating within-block fluctuations as noise rather than signal.

Temporal granularity is further degraded by discarded volumes (three to eight “dummy” scans at run onset) and low-pass filtering choices: study 26 applied a 1.5 s low-pass filter to suppress residual motion, inadvertently removing any neural oscillations above 0.67 Hz—including the chewing rhythm itself. Meanwhile, the typical total run length is short (≈4–6 min), yielding at most 8–10 chewing–rest cycles; this curtails statistical efficiency and hampers time-resolved connectivity or state-transition analyses.

Finally, none of the papers attempted time-frequency or dynamic–functional–connectivity analyses that could have extracted transient coupling between the jaw motor cortex, cerebellum, insula, and brainstem during the switch from quiescence to active mastication. The consequence is a literature that maps where chewing activates the brain, but remains blind to how fast and in what sequence those activations unfold. Upgrading to multi-band EPI with TRs ≤ 800 ms, adopting event-related or variable-epoch designs, and integrating EMG-triggered finite-impulse models are essential steps toward capturing the true temporal choreography of human mastication.

#### 6.1.5. Analytical Scope

The analytical toolkit applied to gum-chewing fMRI remains largely rooted in first-generation univariate statistics. Eleven of the fifteen papers rely almost exclusively on a canonical-HRF general-linear model with box-car regressors that contrast whole chewing blocks against rest. Within this group, nine threshold their statistical maps at uncorrected voxel-wise *p* < 0.001 (studies 26, 27, 28, 29, 30, 31, 32, 35, 36), two are even looser (*p* < 0.01 or 0.05; studies 29, 34), and only three implement any form of family-wise or false-discovery correction (studies 30, 37, 38). Cluster-extent criteria vary from five to twenty voxels, but justification is seldom provided, raising the likelihood of inflated false-positive rates and hindering meta-analysis.

Network-oriented methods are the exception rather than the rule. Psychophysiological interaction (PPI) mapping appears once (study 34) to explore stress-related coupling between anterior insula and dACC; dynamic-causal modelling (DCM) is also confined to that single paper. Independent-component analysis is featured in just two studies: a modest spatial ICA to denoise chewing artefact (study 26) and a full group-ICA decomposition to identify sensorimotor and cognitive components (study 39). No investigation applies seed-to-whole-brain functional connectivity, graph-theoretic metrics (degree, efficiency, modularity), sliding-window dynamic connectivity, or multivariate-pattern analyses (MVPA) that could decode bolus hardness, laterality, or cognitive load from distributed voxel patterns.

In terms of nuisance modelling, the norm is to include the six rigid-body motion parameters as regressors; only one protocol (study 37) augments these with scrubbing of high-motion TRs, and none incorporate physiological noise regressors—even though cardiac and respiratory artefacts are pronounced in infratentorial slices. Parametric modulators tied to behaviour (e.g., bite-force amplitude, inter-bite interval, EMG power, salivary flow, stress rating) are entirely absent, so the field cannot yet map graded neural responses to quantitative aspects of mastication.

Region-of-interest (ROI) frameworks are under-utilised: aside from handedness and chewing-side preference studies that extract beta values from bilateral MI/SI (studies 27, 33), most work reports only peak coordinates or cluster sizes, foregoing systematic ROI analyses that would enable cross-protocol replication. Structural–functional integration is similarly rare; only the ICA paper (study 39) overlays functional components on diffusion templates, and no study correlates white-matter integrity of trigeminal or callosal tracts with activation strength or laterality.

Longitudinal or plasticity-sensitive metrics are missing. The one perfusion study (ASL, study 40) compares pre- vs. post-chewing CBF in brainstem nuclei but stops short of relating flow changes to BOLD connectivity or behavioural gain. No group models within-session adaptation of BOLD amplitude over successive chewing bouts, nor do they examine learning-related shifts in network topology.

In short, the present analytical repertoire answers the binary question “Where is chewing louder than rest?” but remains blind to how distributed circuits co-operate, how fast they reconfigure, and how neural signals scale with masticatory kinematics or cognitive context. Adoption of contemporary multivariate and network analyses—together with richer behavioural regressors and rigorous multiple-comparison control—is imperative for extracting mechanistic insight from the next generation of chewing-fMRI data.

#### 6.1.6. Behavioural and Physiological Coupling

In most gum-chewing fMRI experiments the link between what the brain is doing and what the jaw is actually doing is only assumed, not demonstrated. Of the fifteen studies reviewed, just four captured any real-time peripheral signal. Two papers recorded surface EMG from the masseter or temporalis muscles (one during concurrent hand-movement blocks, the other during soft- versus hard-bolus chewing), but the resulting burst patterns were used merely to confirm task compliance rather than being time-locked to the fMRI design or entered as parametric modulators. A third study monitored bilateral masseter EMG during a one-hour chew, yet this was performed outside the scanner and served solely as a pacing check, while a fourth attached an accelerometer to the head coil to cull motion-contaminated volumes without retaining the trace for jaw-cycle timing.

Objective measurement of bite force—the variable most plausibly driving hardness-dependent BOLD differences—was almost non-existent. No protocol deployed an MRI-compatible force transducer or pressure-sensitive bolus to capture cycle-by-cycle variability. Jaw kinematics fared little better: one study used a kinesiograph to verify that supine chewing resembled normal mastication, but these traces were collected in a separate, seated session and never integrated with the imaging model. The remaining investigations relied on metronome prompts or verbal instruction, leaving unverified whether participants maintained the prescribed rhythm, amplitude, or laterality once the scan commenced.

Concurrently, autonomic and respiratory monitoring was entirely absent, even though chewing modulates sympathetic tone and is tightly coupled to breathing—factors that introduce signal fluctuations in insular and brainstem regions that cannot be regressed without physiological traces. Behavioural or subjective outputs were equally sparse: only one study gathered stress ratings, another collected reaction times and accuracies from an Attentional Network Test, and a third logged psychophysical intensity and lateralisation scores for minty odours; none treated these measures as trial-wise regressors. Swallowing events—which impose abrupt brainstem and cerebellar activations—went untracked, and salivary flow or cortisol assays, despite their relevance to gustatory and stress circuitry, were never sampled.

The consequence is an elegant but incomplete picture: we know where chewing lights up the brain, yet we cannot specify which aspect of the act—force, rate, muscle work, flavour intensity, autonomic arousal, or cognitive set—drives which neural component. Future protocols must integrate MRI-safe bite-force sensors, fibre-optic jaw trackers, high-density EMG, cardiorespiratory belts, swallow microphones, and time-stamped behavioural tasks if they are to transform descriptive activation maps into mechanistic brain-behaviour models of human mastication.

#### 6.1.7. Longitudinal and Translational Relevance

Every chewing-fMRI paper published so far is essentially a snapshot: participants chew for a few minutes (block paradigms totalling 3–7 min inside the scanner) or, in the single ASL perfusion study, for one hour immediately before the post-scan. None return for follow-up imaging days or weeks later, none include repeated baseline sessions to gauge test–retest reliability, and none track whether neural effects persist, habituate, or potentiate with continued chewing practice. Acute designs therefore cannot inform on neuroplastic trajectories—a striking omission given behavioural evidence that weeks of gum use can sharpen attention, relieve stress, or modify trigeminal sensitivity.

Because plasticity is unmeasured, causal interpretation veers into speculation. Papers often extrapolate from a one-off BOLD increase in prefrontal or cerebellar regions to claims about cognitive enhancement or stress mitigation, yet without longitudinal data we cannot know whether such activation reflects transient arousal, novelty, fatigue, or durable circuit strengthening. The field likewise lacks dose–response mapping: no study compares one chewing bout per day versus fifteen, or soft versus hard-diet regimens across months, so the minimum effective dose for neural change remains unknown.

On the clinical-translation front, evidence is even thinner. Despite repeated suggestions that mastication training might aid ageing cognition, no study tests patients, no randomised controlled trials manipulate chewing over time, and no protocol pairs imaging with functional outcomes such as memory scores, articulation speed, nutrient intake, or pain reduction. The single ageing paper provides cross-sectional hints of compensatory prefrontal up-regulation, but without intervention data we cannot tell whether chewing exercises restore youthful activation patterns or merely tax already stretched executive resources. Similarly, the stress-attenuation study demonstrates an acute blunting of anterior-insula reactivity, yet it does not reveal whether habitual gum use recalibrates the stress network or if the effect wanes with familiarity.

Another translational gap concerns vascular health: the ASL study that found a right-sided trigeminal-nucleus perfusion boost did not examine peripheral endothelial function, cerebral autoregulation, or migraine frequency, leaving unanswered whether repeated mastication constitutes beneficial “vascular exercise.” Nor does any paper combine fMRI with structural measures like cortical thickness, diffusion indices, or myelin-sensitive contrasts that could chart long-term tissue change.

Finally, ecological validity is sacrificed by testing chewing in an MRI bore divorcing it from real-world contexts—meal consumption, social interaction, gum flavours, satiety signals—yet no longitudinal naturalistic protocols deploy portable neuroimaging or ecological momentary assessment to bridge the laboratory and life.

In sum, without multi-session, weeks-to-months interventions that integrate behavioural endpoints and patient cohorts, current work tells us little about whether or how chewing shapes brain networks over time or can be harnessed therapeutically. Establishing longitudinal RCTs, embedding imaging within rehabilitation protocols, and pairing neural metrics with clinical outcomes are the indispensable next steps for real translational impact.

### 6.2. fNIRS Studies

The current corpus of fNIRS mastication studies, while internally consistent, is circumscribed by several methodological, sampling, and conceptual constraints that must be remedied before firm practical recommendations can be issued. All experiments to date have relied on small cohorts of neurologically intact, right-handed adults in their twenties or early thirties; no data exist for children whose masticatory apparatus is still developing, for older adults who often experience diminished occlusal force, or for clinical populations such as stroke survivors, dementia patients, chronic-pain sufferers, or individuals with temporomandibular disorders. Expanding the age range, balancing handedness, and including groups with compromised chewing or atypical prefrontal physiology are essential steps if we are to judge the generalisability of the observed haemodynamic and behavioural benefits.

A further limitation is the heterogeneity of chewing protocols. Frequencies have ranged from 30 to 110 cycles min^−1^, bout lengths from 30 s to 20 min, and gum hardness, flavour, and odour have varied idiosyncratically across laboratories. Without a standard reporting framework—stating cadence, side, bite force, texture profile, and moisture absorption—dose–response relationships cannot be modelled and meta-analytic synthesis remains out of reach. Likewise, most investigations have limited optical coverage to the frontal scalp, leaving insular, limbic, and brain-stem structures uncharted even though these regions orchestrate gustatory, affective, and autonomic processing. The adoption of high-density diffuse optical tomography, time-resolved fNIRS, or combined fMRI-fNIRS approaches would afford deeper and more complete neuroanatomical mapping.

Although several groups have used dual-distance optodes to regress scalp and muscle signals, others have relied solely on careful probe placement. Even minor lateral displacement onto the anterior temporal muscle can contaminate cortical channels, as shown experimentally, so future studies should incorporate short-separation or time-gated detectors as a matter of routine and publish quantitative signal-to-noise metrics and control conditions that isolate superficial artefacts. Sampling rates below 4 Hz also risk aliasing the <2 Hz “chewing harmonics” into the haemodynamic band, underscoring the need for faster acquisition or rigorous low-pass filtering.

Conceptually, the field has yet to establish causality between cortical activation and the behavioural outcomes it accompanies. Faster Stroop performance, improved affect under stress, and hypoalgesia all co-occur with PFC hyper-oxygenation, but no study has manipulated prefrontal excitability directly. Integrating mastication paradigms with transcranial magnetic or electric stimulation, or with pharmacological probes, could reveal whether the haemodynamic response is necessary, sufficient, or merely epiphenomenal. Neurochemical profiling has likewise been fragmentary: one study documented a rise in blood serotonin, but adrenergic, dopaminergic, and endocannabinoid pathways—each plausible mediators of mood, vigilance, and pain—remain unexplored. Parallel microdialysis in animal models, plasma metabolomics in humans, and eventually PET-NIRS hybrids would enrich our understanding of the molecular cascades triggered by chewing.

Finally, the extant literature is short-term. The longest human protocol followed participants for only thirty minutes post-chew; no work has examined whether daily gum-chewing practice yields durable gains in executive function, mood or pain tolerance, or whether structural or functional plasticity accrues in masticatory or prefrontal circuits. Longitudinal, randomised trials—ideally in schoolchildren, older adults, and clinical cohorts—are needed to determine the persistence, transferability, and clinical relevance of the acute effects now well documented. Combining wireless high-density fNIRS with wearable autonomic sensors would allow naturalistic tracking of chewing bouts at home, at work or during exercise, linking real-world mastication to fluctuations in mood and performance. In parallel, mechanistic work in rodents, using optogenetic inhibition of trigeminal or ventromedial prefrontal pathways during rhythmic jaw movement, could disentangle bottom-up sensorimotor drive from top-down emotional control. Factorial human experiments that systematically vary flavour valence, odour intensity, chewing cadence, and concurrent cognitive load will ultimately support “personalised mastication”, tailoring gum properties and chewing regimens to the specific goal—heightened focus, stress relief, or analgesia.

By addressing these methodological weaknesses, broadening participant profiles and adopting multimodal, longitudinal, and mechanistic designs, future research can move the field from proof-of-principle to evidence-based prescription, clarifying when, how, and for whom chewing gum constitutes a viable tool for cognitive enhancement, affect regulation, and pain management.

### 6.3. EEG Studies

The present evidence base is constrained first by sample size and composition. All seven EEG studies relied on small convenience cohorts—typically between nine and forty young, healthy volunteers, with a noticeable male bias and virtually no representation of older adults or clinical populations. Such narrow sampling limits statistical power and curtails generalisability; it remains unclear whether the electrophysiological effects of mastication would scale similarly in women, in habitual gum-chewers, in ageing brains, or in people with heightened anxiety and stress reactivity. Future research should therefore recruit larger, demographically balanced groups and explicitly stratify by age, sex, chewing habits, and baseline affective traits in order to capture the true inter-individual variance in chewing-related neurophysiology.

Second, chewing protocols differed so widely that direct comparison is difficult. Durations ranged from one to five minutes, chewing pace was either self-selected or loosely instructed, and gum formulations varied unpredictably in base, sugar, and flavour. Control conditions were equally heterogeneous—stretching from “no gum” to isolated sucrose ingestion or mere aroma inhalation—while participant blinding was never attempted. This procedural scatter makes it impossible to disentangle the mechanical act of mastication from gustatory and olfactory stimulation or caloric load. A logical next step is to adopt a factorial dosing matrix that manipulates mastication (chew versus sham), sweetener type (none, sucrose, non-nutritive), and flavour/aroma (none, mint, citrus) under matched texture and energy conditions; only such systematic control can reveal the specific ingredients that drive EEG change.

A third limitation lies in the EEG methodology itself. Most studies employed sparse 10–20 montages (2–19 electrodes) at modest sampling rates and, in several cases, halted recording during the actual chewing epoch to avoid movement artefacts. The result is coarse spatial resolution and a dearth of information about real-time cortical dynamics. Continuous high-density acquisition (≥64 active channels) combined with jaw EMG or inertial sensors and modern artefact-subtraction pipelines—independent component analysis, wavelet thresholding, adaptive filtering—would permit genuine “online” monitoring of brain activity while participants chew. In parallel, the analytical focus has remained anchored on simple band-power averages; measures of connectivity, cross-frequency coupling, microstate structure, or information-theoretic complexity have scarcely been explored. Incorporating such network-level and dynamical metrics, and corroborating them with complementary haemodynamic imaging (fNIRS or fMRI) and autonomic indices (heart-rate variability, skin conductance), will help establish whether chewing reorganises large-scale brain systems or merely tweaks local oscillatory gain.

Finally, the behavioural canvas has been narrow. Apart from one vigilance paradigm and two stress-induction experiments, no study has probed memory, executive control, or the persistence of mood change beyond a few minutes. Translational value would be greatly enhanced by embedding chewing in tasks that span sustained attention, working memory, and emotion regulation, with follow-up testing over tens of minutes or hours, and by moving experimentation into ecologically valid settings such as classrooms, open-plan offices, long-haul driving simulators, or shift-work environments. Only when EEG signatures are linked robustly to meaningful performance or safety outcomes will it be possible to decide whether chewing gum is merely a fleeting arousal trick or a practical tool for cognitive enhancement and stress mitigation.

### 6.4. Control of Psychological and Physiological States

Many neuroimaging studies on gum chewing suggest potential modulations of stress, arousal, and mood, yet relatively few rigorously assess, or control participants’ baseline psychological and physiological states. Variables such as sleep quality, circadian rhythms, caffeine intake, or even subtle anxiety can modulate neural activity independently of chewing, thus confounding the interpretation of results. Moreover, subjective stress and mood ratings are often collected without standardised or validated scales, limiting the precision with which researchers can link gum-induced neural changes to real shifts in emotional state. Physiological indicators of stress or arousal—such as cortisol levels, skin conductance, or heart rate variability—are similarly underreported, despite their capacity to clarify whether observed neural changes align with genuine shifts in autonomic balance.

Another factor is the variability in participants’ motivational or attentional engagement. Some protocols impose demanding cognitive tasks (like vigilance or arithmetic challenges) while chewing, but others allow free chewing in a low-engagement context. Disentangling the effects of mastication from the broader psychological context (e.g., how challenged or relaxed participants feel) requires protocols that carefully define and standardise mental workload. For example, if a study examines gum chewing under time pressure or fatigue, it should document pre-task stress levels and continuously monitor participants for signs of mental exhaustion or decreased effort. Without rigorous baseline assessments of mood, stress, or alertness—ideally followed by repeated measurements over the course of the experiment—researchers cannot be certain which portion of the neural or behavioural changes observed arise from chewing as opposed to preexisting fluctuations in arousal.

Methodologically, within-subject designs offer a strong approach to controlling for inter-individual differences in psychological or physiological states. By having each participant undergo multiple conditions (chewing vs. non-chewing, or different gum flavours/aromas) in counterbalanced order, investigators can statistically isolate the gum effect from idiosyncratic traits like trait anxiety or typical daily caffeine use. In addition, randomising the sequence of tasks or flavour exposures helps reduce the impact of learning, boredom, and diurnal variations in stress hormones. More advanced setups might monitor participants’ heart rate variability or electrodermal activity in real time to confirm that each condition elicits consistent or predictable changes in autonomic responses. Finally, transparent reporting of key variables—such as participants’ last meal, recent exercise, and any relevant medical or psychological history—allows for clearer cross-study comparisons. By systematically controlling and measuring these psychological and physiological factors, future research can better delineate the true neural and cognitive effects of gum chewing from the broad array of baseline or task-related influences on brain function.

### 6.5. The Unresolved Question—How Long to Chew Gum to Cause Lasting Changes in the Brain?

As the studies in this review indicate, gum chewing produces immediate neural effects detectable by different neuroimaging techniques. However, none of the studies measured the lasting effects of gum chewing, for example, by comparing brain activity before chewing and after regular chewing for several weeks. For this reason, it remains unclear whether gum chewing can produce long-term changes in brain activity. This highlights the need for future studies to investigate the effects of sustained gum chewing, particularly in people experiencing depression, anxiety, or stress.

Research on the effects of gum chewing presents unique challenges in control group selection and study design. Creating a sham gum chewing control group is inherently difficult. To ensure comparability, the control arm must either chew gum or perform the chewing reflex. The most feasible solutions for this involve between-group study designs comparing different types of chewing gum (e.g., unflavoured vs. flavoured), and to include a control group that performs chewing movements without gum in order to isolate the effects of long-term gum chewing on brain activity.

### 6.6. Gum Chewing—Safety Issues

Another important aspect to consider is the safety of long-term gum chewing. In behavioural studies that did not involve neuroimaging techniques, participants were instructed to chew gum at different frequencies; for example, daily for half an hour over 19 days [17] or twice a day for five minutes over two weeks [18]. These studies reported non-adverse side effects from regular gum use. However, it is imperative to acknowledge that a broader body of research, spanning four decades, indicates that the seemingly innocuous habit of chewing gum can produce a cluster of peripheral side-effects. Many of these are well-documented in peer-reviewed literature and possess plausible pathways for influencing brain activity.

Repeated, forceful mastication can overload masticatory muscles and the TMJ. A BMC Oral Health experiment asked healthy adults to chew hard gum for 40 min; within hours, participants developed significant jaw-muscle pain and fatigue that mimicked painful TMD, confirming a causal overload model [151]. Reinforcing this, a 2025 systematic review of eight epidemiological studies reported a clear dose-response: individuals who chewed gum for ≥60 min day^−1^ exhibited markedly higher odds of developing arthralgia, myofascial pain, and masseter hypertrophy. Importantly, these symptoms often subsided when the chewing habit stopped [152]. Furthermore, degenerative ageing changes render older jaws less tolerant of chronic parafunction, a point emphasised in a narrative gerodontology review linking age-related cartilage thinning to increased susceptibility to masticatory overuse injuries [153]. From a neural perspective, nociceptive input from the trigeminal system is known to modulate thalamo-cortical oscillations, suggesting chronic jaw pain can indirectly reshape EEG rhythms [154].

Chewing activates cephalic-phase reflexes [155]. In duodenal-ulcer patients, seven sticks of gum chewed for 15 min released gastric acid to 36% of the pentagastrin maximum—a response almost identical to that observed with chewing and spitting real food [156]. Furthermore, a 2015 meta-analysis of four peri-operative RCTs confirmed a small but significant increase in fasting gastric fluid volume (≈0.21 mL kg^−1^) after gum chewing, although pH remained unchanged [157]. Even modest shifts in gastric pH or volume can transmit visceral discomfort signals to the insula and anterior cingulate [158], both brain regions known to generate slow EEG rhythms [159,160].

Most short-term crossover trials show that 30–60 min of gum chewing suppresses subjective hunger and snack cravings. One laboratory study found a significant reduction in rated appetite and cravings after 45 min of mastication [161]. A 2025 PRISMA systematic review of nine RCTs supported these findings: five reported significant hunger suppression, and three noted a reduced desire for sweet snacks, although effects on total energy intake and body weight were inconsistent [162]. These observed behavioural changes are likely mediated by appetite hormones (e.g., GLP-1, ghrelin), which interact with hypothalamic and limbic networks [163,164]. This hormonal modulation offers a plausible link between chewing-induced metabolic signals and the EEG changes noted in vigilance experiments.

Food-additive hypersensitivity is a recognised, though uncommon, risk. Two recent case reports have documented IgE-mediated anaphylaxis triggered by guar-gum–containing sweets, both successfully reversed with epinephrine [165]. Cinnamon-flavoured gums are an even more frequent culprit: multiple case reports, along with a 40-patient series, describe allergic contact stomatitis presenting as burning mucosal patches that resolve once the gum is eliminated [166,167]. Systemic allergic reactions release cytokines [168] which can transiently alter cortical arousal patterns.

Beyond hypersensitivity, developmental and physiological factors in children warrant consideration, as their dentitions and digestive tracts are still developing. A 2021 systematic review found that primary-dentition children with malocclusion already exhibit reduced masticatory efficiency; habitual gum chewing could accentuate asymmetric loading and muscle over-activation during critical growth periods [169]. Furthermore, sugar-free gums add osmotic sugar-alcohols that can provoke diarrhoea in sensitive youngsters or irritable-bowel patients [170].

## 7. Societal and Scientific Justification for Research on Gum-Induced Brain Activity

This review received no funding, materials, or other support from chewing gum manufacturers, nor does it endorse any specific brand. Chewing gum is discussed solely as a practical experimental tool: it is hygienic, calorie-neutral, uniform in texture and flavour, and can be easily initiated or halted within a single fMRI, EEG, or fNIRS epoch. This allows for precise time-locking of rhythmic jaw-muscle activity to neurophysiological recording windows—an advantage unattainable with ordinary meals or inert paraffin wax. Thus, the focus on gum chewing reflects methodological tractability, not commercial intent.

From a public-health perspective, understanding the brain mechanisms engaged by brief bouts of mastication is critically important. Globally, 55.2 million people live with dementia, a figure projected to triple by 2050. In 2019 alone, the economic burden reached USD 1.3 trillion—half of this cost borne by unpaid family carers [171]. Large cohort studies and meta-analyses now implicate tooth loss and other markers of masticatory dysfunction as independent, dose-responsive risk factors for cognitive decline and all-cause dementia [172]. Pre-clinical research further supports a causal connection: young rodents on a nutritionally adequate but soft diet exhibit hippocampal atrophy, reduced brain-derived neurotrophic factor, and impaired spatial memory—all of which can be reversed if chewing is reintroduced before mid-adolescence [173,174,175]. These converging lines of evidence suggest that diminished oral-somatosensory input is not a benign consequence of modern food processing but a modifiable contributor to brain ageing.

Chewing gum offers an ethically and logistically straightforward way to re-introduce that missing sensorimotor drive in human experiments. Randomised, double-blind trials show that ten minutes of gum chewing improves sustained attention and reduces commission errors on digit-vigilance tasks, with residual benefits persisting for at least an hour after the gum is discarded. Longer field interventions in office staff report higher self-rated productivity, fewer cognitive lapses, and an attenuation of the typical mid-afternoon dip in alertness, effects accompanied by favourable changes in heart-rate variability and salivary cortisol. Neuroimaging studies utilising fNIRS and high-density EEG reveal that these behavioural gains coincide with dynamic modulation of prefrontal and sensorimotor cortices, limbic structures, and cerebellum. Emotional valence and bite force each tune the magnitude of the haemodynamic response, underscoring the paradigm’s richness for hypothesis-driven work. Mapping such patterns is not a marketing exercise—rather, it is a prerequisite for quantifying dose–response curves (e.g., how hard, how often, how long to chew) and for identifying biomarkers that can track the efficacy of any future mastication-based interventions.

The potential translational dividends of this research are wide-ranging. In paediatrics, adequate chewing stimulation during critical neurodevelopmental windows may support executive function and language acquisition. In middle age, scheduled gum chewing could serve as a low-cost stress-regulation strategy in high-demand workplaces. For older adults, maintaining occlusal load—via natural dentition, prosthodontic design, or gum-mediated training—might help slow cognitive frailty and reduce dementia incidence. Rehabilitation medicine already exploits similar principles: post-stroke patients perform repetitive, resistance-based limb movements to re-engage deprived motor circuits; rhythmic mastication may play an analogous role for cranio-cerebral pathways. Clarifying these neural mechanisms now will enable clinical protocols to be built on measurable biomarkers rather than intuition.

Moreover, the resource footprint of this research agenda is minimal. EEG caps and fNIRS headbands are standard in cognitive laboratories; adding a gum-chewing block extends an experiment by mere minutes and costs only a few cents per participant—orders of magnitude cheaper than pharmacological or neurostimulation challenges, and with negligible safety or regulatory barriers. Given the trillion-dollar global cost of dementia and the rising prevalence of stress-related mood disorders, investing modest scientific resources to understand how a readily available, behaviour-based stimulus modulates brain function is not indulgent: it is a proportionate, strategic, and potentially high-yield approach.

Taken together, the epidemiological burden, the mechanistic plausibility grounded in animal work, the encouraging early human data, and the low incremental cost all justify continued investigation of the neurophysiological correlates of gum-evoked mastication. This work aims to inform dietary guidelines, dental-prosthesis engineering, and neurorehabilitation—not to promote supermarket sales of flavoured confections.

## 8. Conclusions

Accumulating evidence from fMRI, fNIRS, and EEG research underscores that chewing gum is more than a trivial oral habit. Across modalities, studies consistently show that mastication robustly activates the sensorimotor network—including bilateral motor and somatosensory cortices, the cerebellum, and the insula—while also engaging brain regions implicated in stress regulation, emotional processing, and cognitive control. Despite variability in methodologies making direct comparisons challenging, the findings collectively indicate that chewing gum can momentarily boost alertness, modulate stress responses, and potentially activate networks linked to memory function. These effects appear to be driven not just by the mechanical act of mastication but also by factors such as gum flavour, chewing speed, and individual preferences. Notably, the included studies using various neuroimaging techniques seldom incorporated behavioural and cognitive assessments capable of linking observed neural processes during gum chewing to functional outcomes in participants. By contrast, separate behavioural studies—lacking neuroimaging—have demonstrated positive effects. Therefore, a critical question remains unanswered—does gum chewing permanently change brain activity, as measured by fMRI, fNIRS and EEG, which could contribute to improvements in functional outcomes? It is possible that these neuronal changes occur only for the duration of gum chewing and are not permanent. Currently, we cannot confirm whether neural changes induced by gum chewing or if they reflect behavioural gains.

Nonetheless, many of the observed benefits—whether improved reaction times, reductions in perceived stress, or shifts in EEG power—tend to be transient, underscoring the need for additional research on duration and dosage effects. Methodological refinements, such as careful control of flavour and odour confounds, higher-resolution imaging of brainstem and cerebellar pathways, and rigorous artifact reduction protocols, will be crucial for clarifying the neural mechanisms at play. Equally important is exploring the possibility of longer-term cognitive or stress-regulatory benefits through longitudinal studies. Ultimately, while chewing gum is an accessible, low-cost behaviour, the growing body of neuroimaging data may suggest it holds meaningful potential for enhancing cognitive performance, emotional resilience, and momentary attentional states. With more rigorous and targeted research, the simple act of chewing could emerge as a valuable adjunct to strategies aimed at boosting mental performance and managing stress in everyday life.

## Figures and Tables

**Table 1:** fMRI studies included in the review.

Reference	Sample Characteristics	Experimental Paradigm	Main Findings
[26]	17 (10M, 7F, aged 20–31)	Chewing moderately hard vs. hard gum (1 Hz)	Bilateral activation in M1, S1, SMA, insula, thalamus, cerebellum; SMA and insula more active with moderately hard gum; cerebellum more active with hard gum.
[27]	16 (10F, 6M, aged 23–38)	Chewing based on chewing-side preference	Contralateral SI/MI activation; left CSP → right substantia nigra; right CSP → left cerebellum; activation in IFG, IPL, and left insula.
[28]	9 (6M, 3F, aged 22–31)	Finger movement with and without chewing	Reduced SM1 activation when chewing added (1296 vs. 2090 voxels); significant decrease in M1 and S1 activation.
[29]	8 (4M, 4F, aged 20–29)	Chewing tasteless gum on right molars	Activation in left hippocampus, entorhinal cortex (BA28), and parahippocampal cortex (BA36); no significant perirhinal cortex activation.
[30]	15 (6F, mean age 25.3)	Unilateral chewing (left and right)	Bilateral activation in S1, S2, M1, SMA, cingulate, insula, thalamus, cerebellum; symmetrical patterns; lateralisation index < 0.055.
[31]	29 (15M, 14F, mean age 24.0)	Right-side chewing, segmental timing analysis	Activation in M1, SMA, cerebellum, caudate, cingulate gyrus; early chewing → frontal regions; later chewing → cerebellum and superior temporal gyrus.
[32]	12 (6M, 6F, aged 20–28)	Gum chewing vs. sham chewing	SMA, PMA, insula, SPL, frontal and parietal lobes more active in real chewing than sham chewing.
[33]	10 (5R, 5L-handed, ~28.2 years)	Chewing soft vs. hard bolus in varied posture	Right-handed → right hemisphere dominance; left-handed → left hemisphere; soft bolus activated more cortical areas; bilateral SMA, M1, S1, Broca’s, insula.
[34]	16 (6F, mean age 22.7)	Stress (noise) with/without chewing	Chewing reduced activation in STS and AI during noise; reduced AI–dACC connectivity; gum disrupted transmission of stress-related signals.
[35]	17 (8F, aged 20–34)	Attention Network Task with/without chewing	Chewing improved reaction time; increased ACC and frontal gyrus activity; anterior cerebellum showed reduced activity during chewing.
[36]	29 (13F, mean age 23)	Odor perception in gum users vs. non-users	High-frequency gum users → more trigeminal activation (midcingulate, SMA, pre/postcentral); low-users → more hippocampus and OFC activity.
[37]	32 (11M, 21F, aged 18–50)	Spontaneous vs. controlled chewing + rosary task	Cerebellum activated during rhythmic chewing and rosary pulling; not during voluntary chewing; M1, S1, and premotor areas activated in all chewing tasks.
[38]	27 (3 age groups: YA, MA, EA)	Gum chewing across age groups	M1, thalamus, cerebellum activation decreased with age; right prefrontal activation increased with age; no age effects in SMA or insula.
[39]	38 (from 60, aged 18–35)	Natural chewing vs. rest using Group ICA	ICA revealed 3 networks: (1) sensorimotor, (2) cognitive–emotional (ACC, BA9/10), (3) syntax-related (IFG, BA47); chewing engages multiple functional systems.
[40]	18 (9M, 9F, aged 19–28)	1 h chewing with ASL perfusion MRI	Increased blood flow in right trigeminal nucleus (Vp); correlated with chewing-side preference and masseter muscle volume.

**Table 2:** fNIRS studies included in the review.

Reference	Sample Characteristics	Experimental Paradigm	Main Findings
[41]	36 (19M, 17F, mean age 28)	Chewing palatable vs. unpalatable gum	Higher left DLPFC/frontopolar activation with unpalatable gum; no muscle/HR differences
[42]	25 (13M, 12F, mean age 27)	Chewing 3 gums (C, T, TO); TCD + NIRS	TO-gum → ↑ DO2Hb and MCAV; effects lasted 2 min post-task; no EMG differences
[43]	25 (11M, 14F, mean age 27.3)	Free vs. controlled chewing (right side, rhythm)	↑ MCAV during all chewing; no differences between conditions; muscle activation varied
[44]	8 (mean age 25.3)	Left-side gum chewing; dual-distance probes	↑ Total-Hb in PFC; superficial muscle activity affected signals in deviated probe
[45]	14 (7M, 7F, mean age 26.9)	Stroop test with/without chewing	↑ Oxy-Hb in left DLPFC with gum; faster RTs, unchanged accuracy
[46]	12 (10M, 2F, mean age 24.0)	Negative sound exposure ± gum	↑ PFC activation with gum; enhanced alpha waves; ↑ HR; reduced discomfort
[47]	11 (9M, 2F, mean age 26.8)	Negative sound exposure ± gum	↑ PFC activation and alpha waves with gum; ↓ STAI anxiety, ↑ VAS comfort
[48]	11 (9F, 2M, mean age 20.9)	Chewing at 3 speeds (30, 70, 110 CPM)	↑ Oxy-Hb with chewing; highest at 110 CPM; ↓ Deoxy-Hb; no change in total-Hb
[49]	11 (9F, 2M, mean age 20.5)	Uchida-Kraepelin test + chewing (3 speeds)	↑ Oxy-Hb at 110 CPM; no cognitive performance improvements
[50]	11 males (mean age 29.5)	Gum + walking + pleasant sounds	↑ PFC activity in gum and walking + gum; ↑ pleasantness in VAS
[51]	10 (5M, 5F, aged 26–37)	20 min chewing; serotonin + pain analysis	↑ Oxy-Hb in ventral PFC; ↓ pain reflex; ↑ blood serotonin; no deoxy-Hb change
[52]	30 (11F, 19M, mean age 23.7)	Serial recall task pre/post chewing	↑ Oxy-Hb during chewing; no change in recall accuracy or reaction time

**Table 3:** EEG studies included in the review.

Reference	Sample Characteristics	Experimental Paradigm	Main Findings
[46]	12 healthy adults (10M, 2F; mean age 24.0 years)	Block-design eyes-closed listening to unpleasant IADS-2 sounds without gum (NS) versus with tasteless gum (NS + Gum)	Alpha-wave appearance rate fell during NS (42.78%) but rose significantly when chewing gum (44.43%; *p* = 0.0227, Cohen d = 0.85)
[47]	11 healthy adults (9M, 2F; mean age 26.8 years)	Identical unpleasant-sound blocks without vs. with gum under eyes-closed resting instructions	Alpha-wave appearance rate increased from 44.00% (NS) to 47.10% (NS + Gum; *p* < 0.05), showing chewing-related attenuation of stress-induced alpha suppression
[53]	40 right-handed adults	Vigilance task with chewing vs. no chewing, 4 sessions (baseline, during, post)	↓ Reaction times, ↑ correct detections during chewing; ↑ beta power (F7, T3) post-chewing; ↑ HR during chewing; alertness maintained; EEG and HR effects were temporary
[54]	20 (11M, 9F), aged 24–34	Chewing gum base, gum + spearmint, gum + sucrose; aroma and ingestion control	Gum base: ↑ beta, ↓ theta → arousal; Spearmint: ↑ beta, ↓ alpha → stimulation; Gum + sucrose: ↑ theta, ↓ beta → relaxation; aroma mirrored spearmint effects; sucrose alone = no change
[55]	11 (7M, 4F), aged 24–32	Resting EEG vs. post-chewing spearmint gum for 3 min	↑ Alpha in most brain regions (O, T, F, P); interpreted as arousal and alertness effect; bilateral activation; no hemispheric asymmetry
[56]	9 (6M, 3F), aged 27–33	Chewing flavoured vs. unflavoured gum; inhaling flavoured oil	Flavoured gum: ↑ alpha and beta, ↓ theta → arousal; Unflavoured gum: ↑ alpha, ↓ beta → relaxation; oil had similar effects as flavoured gum, showing influence of both flavour and mastication
[57]	20 males, mean age 24.9 ± 4.9	Chewing gum base, flavoured gum, theanine gum; EEG source localisation + VAS ratings	Flavoured gum → anterior/right source shift (↑ alpha-2, beta-2); gum base → posterior/left; ↑ Global Field Power for delta-theta, alpha-2, beta-1 after flavoured gum; ↑ refreshment and comfort ratings

## Data Availability

No new data were created or analysed in this study. Data sharing is not applicable to this article.
